# MLViS: A Web Tool for Machine Learning-Based Virtual Screening in Early-Phase of Drug Discovery and Development

**DOI:** 10.1371/journal.pone.0124600

**Published:** 2015-04-30

**Authors:** Selcuk Korkmaz, Gokmen Zararsiz, Dincer Goksuluk

**Affiliations:** Department of Biostatistics, Faculty of Medicine, Hacettepe University, Sihhiye, Ankara, Turkey; University of Minnesota, UNITED STATES

## Abstract

Virtual screening is an important step in early-phase of drug discovery process. Since there are thousands of compounds, this step should be both fast and effective in order to distinguish drug-like and nondrug-like molecules. Statistical machine learning methods are widely used in drug discovery studies for classification purpose. Here, we aim to develop a new tool, which can classify molecules as drug-like and nondrug-like based on various machine learning methods, including discriminant, tree-based, kernel-based, ensemble and other algorithms. To construct this tool, first, performances of twenty-three different machine learning algorithms are compared by ten different measures, then, ten best performing algorithms have been selected based on principal component and hierarchical cluster analysis results. Besides classification, this application has also ability to create heat map and dendrogram for visual inspection of the molecules through hierarchical cluster analysis. Moreover, users can connect the PubChem database to download molecular information and to create two-dimensional structures of compounds. This application is freely available through www.biosoft.hacettepe.edu.tr/MLViS/.

## Introduction

Discovery and development of a new drug can be simply divided into four steps: (i) target identification, (ii) lead finding and optimization, (iii) pre-clinical studies and (iv) clinical studies. Discovery of new drug candidates is becoming increasingly hard, costly and time-consuming. This process can take between 12–15 years and cost over one billion dollars. Many efforts have been made to decrease the cost and time, and increase the effectiveness of this process [1,2]. In the early-phase of this process, there are thousands of compounds in the chemical libraries. Virtual screening methods, which are fast, effective and comparatively cheap, can be used to evaluate these compounds in the early step of drug discovery and development studies. These methods can be divided into two parts as structure-based and ligand-based approaches. Structure based approaches predict conformation of the ligands within the active site of target macromolecule, while ligand-based approaches predict active molecules in a database with using information about a set of ligands that are known to be active for a given target [3].

Statistical machine learning methods are fast and effective algorithms and widely used in various fields, including drug discovery, structural biology and cheminformatics. Since these methods can deal with high-dimensional data, they are suitable for virtual screening of large compound libraries to classify molecules as active or inactive or to rank based on their activity levels. In the literature, there are many studies that explore the performances of these methods in the early-phase of drug discovery and development. These studies mainly focused on two parts: classification and activity prediction of molecules. For classification task, Korkmaz *et al*. [4] used support vector machines (SVM) incorporating with several feature selection methods to classify molecules as drug-like and nondrug-like whereas Garcia-Sosa *et al*. [5] performed a logistic regression on the same set of data. Byvatov *et al*. (2003) [6] and Zernov *et al*. [7] compared performances of SVM and neural networks (NN) on drug-like/nondrug-like classification problem and they both concluded that SVM outperformed NN. Moreover, SVM used to classify certain kind of inhibitors, such as butyrylcholinesterase [8], lymphocyte-specific protein tyrosine kinase [9] and cytochrome P450 [10]. Other machine learning methods, such as NN [11,12], naïve Bayes (NB) [8,13] and k-nearest neighbor (KNN) [14], have been also applied to distinguish active molecules from inactive ones. For activity prediction, Gertrudes *et al*. [15] compared performances of various machine learning methods in prediction of biological activity of molecules. Jorissen and Gilson [16], Wassermann *et al*. [17], Agarwal *et al*. [18] and Rathke *et al*. [19] used the SVM algorithm to rank molecules based on their activity. Other methods, such as Bayesian neural networks (BNN) [20] and random forest (RF) [21,22], are also used for activity prediction.

In this study, we mainly focused on classification task and our aim was to develop a new tool, which can classify the compounds as drug-like and nondrug-like, for virtual screening of small molecules. For this purpose, we trained a number of machine learning methods and compared their performances based on ten different measures. To find the best performing algorithms, we have made use of principal component (PC) and hierarchical cluster (HC) analyses. Finally, we have developed our application using the best performing machine learning algorithms. Besides supervised methods, such as classification and regression, unsupervised methods, like clustering, are also used in drug discovery studies. Since similar compounds have similar properties, it should be adequate to understand structure-activity relationships of the entire compound set with analyzing the representative compounds from each cluster instead of performing the time-consuming complete set of experiments [23]. Hence, this tool has ability to perform hierarchical cluster analysis and to create heat map and dendrogram for visual inspection of the molecules. Furthermore, researchers can connect the PubChem, which is a database of small molecules and provides information on the biological activities of them, via this tool. It allows users to download structure data file (SDF) of compounds, which contains molecular information about compounds, and to plot two-dimensional structures of molecules. All analyses conducted in R software [24], version 3.1.1, using Rcpi [23], caret [25], shiny [26], gplots [27] and ChemmineR [28] packages.

## Materials and Methods

### Data sets

The data sets (training and test) used in this study are collected from a two recent publication [4,5]. The original data sets retrieved from Garcia-Sosa *et al*. [5], in which the training set contained 631 compounds (311 drug-like and 320 nondrug-like compounds, S1 Table) and an independent test set contained 216 compounds (98 drug-like and 118 nondrug-like compounds, S2 Table). Korkmaz *et al*. [4] used these data sets and applied different feature selection methods, including recursive feature elimination, wrapper method and subset selection, before performing SVM. They found that feature selection methods improved the discrimination ability of the SVM classifier and subset selection outperformed other methods. According to their results, the subset selection method selected six molecular descriptors as the best features, including logP, polar surface area (PSA), donor count (DC), aliphatic ring count (AlRC), aromatic ring count (ArRC) and Balaban index (BI). Here, all descriptors are obtained by a calculation method. The atom-additive XLOGP method is used to calculate the logP and other descriptors are calculated by using Marvin Beans version 5.3.8 [5]. The authors obtained 81% accuracy rate, 88% sensitivity, 75% specificity and 88% area under the curve using SVM algorithm. For our purposes, we used these data sets with the best six features to train and test various machine-learning methods.

### Statistical machine learning methods

To classify compounds in a fast and effective way, we made use of the utility of different statistical machine learning algorithms. Within this scope, we trained various discriminant, tree-based, kernel-based and ensemble classifiers, and some other models including NB, NN, KNN and learning vector quantization (LVQ). In this section, we give a brief overview of these statistical learning models.

Linear discriminant analysis (LDA) is among the most popular classification technique in statistics and pattern recognition. It aims to estimate the posterior probabilities of classes using the density and prior probabilities of the data classes. There are a number of ways to motivate LDA classifier. Bayesian rule is a widely used method for this purpose. Let *X* and *Y* refer to random variables for molecular descriptors and the class label of compounds, and let *f*_*k*_(*x*) be the class-conditional density function and *π*_*k*_ be the prior probability for class *k*. Using Bayes’ theorem, posterior probability of a compound for class *k* is:

$$\mathrm{Pr}\left(C=k|X=x\right)=\frac{f_{k}\left(x\right)\pi_{k}}{\sum_{c=1}^{k}f_{k}\left(x\right)\pi_{c}}$$ (1)

LDA uses multivariate normal distribution as a density function:

fk(x)=1(2π)p2|Σk|12e−12(x−μk)TΣk−1(x−μk) (2)

where *p* is the number of molecular descriptors, *μ*_*k*_ is the sample mean vector, Σ_*k*_ is the sample variance-covariance matrix for class *k*. This matrix contains the pooled variances and covariances of descriptors. Organizing Eq (1) with multivariate normal distribution and performing some algebra, we obtain the linear discriminant function as $\delta_{k}\left(x^{*}\right)=x^{T}\Sigma^{-1}\mu_{k}-\frac{1}{2}\mu_{k}^{T}\Sigma^{-1}\mu_{k}+\log\pi_{k}$ and assign a new test compound to the class that maximizes this function.

Other discriminant classifiers are extensions of LDA. In quadratic discriminant analysis (QDA), each class uses their own covariance matrices rather than using a common one. Robust linear and robust quadratic discriminant analyses (RLDA, RQDA) use robust estimators to estimate mean vectors *μ*_*k*_ and variance-covariance matrices Σ_*k*_. Flexible discriminant analysis (FDA) uses a nonparametric form of linear regression to handle LDA problem. Mixture discriminant analysis (MDA) models the density of each class from two or more Gaussian functions with different centroids. Nearest shrunken centroids (NSC) is a sparse classifier, which is originally developed for microarray data classification. This algorithm shrinks class means to the overall mean for feature selection purpose, and classifies the data with the selected features using diagonal discriminant analysis, which ignores correlations among features [29–31].

Decision trees aim to build a model to extract decision rules to predict the response variable (compound classes) based on features (molecular descriptors). Each interior node represents a descriptor and there are edges to children for each possible descriptor value. Leaf nodes correspond to compound classes for the values of descriptors by the path from the root to the leaf. Classification and regression trees (CART) and J48 (also known as C4.5) are widely used decision tree algorithms that grow the whole tree first and then prune it back to control over-fitting. Even these two algorithms have similar methodologies; there are some considerable differences between them: first, CART allows binary testing while C4.5 allows two or more outcomes, second, CART uses gini index, while C4.5 uses information gain as splitting criteria and third, CART uses cross-validation based cost-complexity model, while C4.5 uses binomial confidence limits based single pass algorithm to prune trees. C5.0 is an extension of J48 algorithm. It is faster, more memory efficient, provides smaller decision trees, allows user to weight cases, winnows the useless features automatically and supports boosting to improve the performance compared to J48. Conditional inference trees (CIT) conducts a significance testing rather than maximizing the splitting criteria (e.g. gini coefficient, information gain), and avoids feature selection bias [31–33].

When the data is linearly non-separable, kernel-based classifiers can be a good choice. Kernel functions, which are used with these classifiers, transform the data to higher dimensions and make linear models work in nonlinear settings. SVM is known among the most popular kernel-based classifiers due to its strong mathematical background, accurate performance and ability for high-dimensional classification. The main objective of SVM is to find the optimal function that maximizes the distance (known as margin) among closest data points in different classes (known as support vectors) to separate the data. SVM use quadratic programming and Lagrange multipliers for this purpose. SVM applies kernel functions including radial-basis function (RBF) and polynomial functions for nonlinear classification problems. Another kernel-based classifier least squares support vector machines (lsSVM) are special cases of SVM, which solves a linear system rather than using quadratic programming in optimizing the model parameters. In both SVM and lsSVM models, we considered linear and radial-basis function as kernels. Partial least squares (PLS) uses principal factors for classification instead of applying original descriptors. These principal factors are the projected lower dimensional versions of descriptors, which explain the maximum variance of the data. PLS applies linear classifiers after the projection process [31,32,34].

Another algorithm that has similar properties with discriminant classifiers is NB. However, unlike discriminant methods, it considers each descriptor independently contribute to class prediction. It also uses Bayes’ theorem to predict the posterior probabilities in order to identify the class label, which the compounds to be assigned. KNN is a lazy learner classifier, where a compound is classified to the class, which is most common in its k-nearest neighbors. Here, input can be considered as *k* closest training data points and output is the class labels. NN is inspired by the brain central nervous system and similarly contains the inter-connected neurons in its algorithm structure. It takes the input data, weights and transforms it with activation functions. Activation is passed from one neuron to other until an output neuron is activated. LVQ is a special case of NN algorithm, which is also related with KNN. It applies a winner-take-all approach and the winner prototype moves close to training samples in its class if it correctly classifies the compound, or moved away if it misclassifies the compound [31].

Instead of fitting a single model, multiple models applied by ensemble algorithms are used to improve the classification accuracy, reduce variance and avoid over-fitting. Bagging is one of the widely used ensemble algorithms. Given a training data set, bagging (also called as bootstrap aggregating) method firstly generates multiple datasets using bootstrap technique, then trains each bootstrap data using a specific classification algorithm and finally aggregates the results of each model with a suitable technique, such as majority voting. RF is the most famous bagging ensemble algorithm, which combines single decision tree models to achieve higher classification accuracy. Accordingly, bagged support vector machines (bagSVM) and bagged k-nearest neighbors (bagKNN) are bagging ensembles of SVM and KNN classifiers [31,32,35,36]. Readers can find further details about these classifiers in referenced papers.

### Model building

Since several classifiers used in this study require the predictor variables centered and scaled [37], first, the training set is centered and scaled using z-score transformation. Then, the test set is centered and scaled based on the parameters (i.e. mean and standard deviation) of the training set. Most of the machine learning methods, which are introduced in the previous section, except LDA, RLDA, QDA and RQDA from discriminant classifiers and lsSVMlin from kernel-based classifiers, include at least one tuning parameter in order to avoid either overfitting or underfitting. Hence, in the training set, we made a grid search and used 10-fold cross-validation to select optimal tuning parameters. We repeated this procedure 10 times to stabilize the test errors and provide more reliable model estimates. All model building steps are applied in caret package version 6.0–35 [25] of R version 3.1.1 [24].

In discriminant classifiers; number of subclasses is set as 6 for MDA, product degree and number of terms are selected as 1 and 14, respectively, for FDA and shrinkage threshold is optimized as 5.89 for NSC. In decision tree classifiers; a rule-based model is used whereas predictor winnowing is not and number of boosting iterations is determined as 50 for C5.0, confidence threshold is set as 0.25 for J48 and complexity parameter is specified as 0 for CART. In kernel-based classifiers; cost parameter is determined as 1 for SVMlin, sigma and cost parameter are set as 0.25 and 4, respectively, for SVMrbf, sigma parameter is set as 0.22 for lsSVMrbf and number of components is identified as 6 for PLS. In ensemble classifiers; number of randomly selected predictors is founded as 2 and 500 trees are used for RF, number of bootstraps is set as 100 for bagSVM and bagKNN, and radial basis function used as kernel for bagSVM. In other classifiers; Laplace correction is selected as 0 and normal density is estimated for NB, number of hidden units and weight decay are optimized as 13 and 0.1, respectively, for NN, number of neighbors are determined as 7 for KNN, and codebook size and number of prototypes are determined as 3 and 1, respectively, for LVQ. We, then, fit the methods to the training set with the selected value of the tuning parameters.

### Performance assessment

To compare the performance of the methods, we calculated various diagnostic measures, including; accuracy rate (AR), sensitivity (SE), specificity (SP), positive predictive value (PPV), negative predictive value (NPV), detection rate (DR), balanced accuracy rate (bAR), F-score (FS), Matthews correlation coefficient (MCC) and Kappa statistic (κ) as follows:

$$AR=\frac{TP+TN}{TP+TN+FP+FN}$$

$$SE=\frac{TP}{TP+FN}$$

$$SP=\frac{TN}{TN+FP}$$

$$PPV=\frac{TP}{TP+FP}$$

$$NPV=\frac{TN}{TN+FN}$$

$$DR=\frac{TP}{TP+TN+FP+FN}$$

$$bAR=\frac{SE+SP}{2}$$

$$FS=2\frac{SE\times PPV}{SE+PPV}$$

$$MCC=2\frac{TP\times TN-FP\times FN}{\sqrt{\left(TP+FP\right)\left(TP+FN\right)\left(TN+FP\right)\left(TN+FN\right)}}$$

$$\kappa =\frac{AR-p_{e}}{1-p_{e}}$$

where, *p*_*e*_ = ((*TP* + *FN*)(*TP* + *FP*) + (*FP* + *TN*)(*FN* + *TN*))/ *n* ^*2*^, *TP* = true positives, *TN* = true negatives, *FP* = false positives and *FP* = false negatives.

To reveal the best performing methods in a more advanced way, we applied PC and HC analyses using performance measures results. For PC analysis, we extracted two components that explain the 99.90% of the total variation, and used varimax rotation to more explicitly differentiate the factor loadings of each variable on a given component. For HC analysis, Euclidean distance metric and Ward method are used to cluster the algorithms used in this study based on their classification successes.

## Results and Discussion

We compared the performances of twenty-three different machine-learning methods based on performance measures and the results are summarized in Table 1. According to these measures, all methods have comparable results. AR obtained between 68%-79%, and lsSVMrbf, FDA and C5.0 were the best performing algorithms among others. SE and SP values were between 81%-92% and 51%-71% respectively, and LDA, NSC and SVMrbf were outperformed other algorithms regarding SE, and SVMrbf, FDA and C5.0 were the top three algorithms based on SP. PPV and NPV results were between 60%-72% and 81%-90% respectively, and lsSVMrbf, C5.0 and FDA were the best performing algorithms for PPV measure, and RLDA, NSC, SVMrbf and PLS had the highest NPV values. DR values were between 37%-42%, RLDA, NSC and PLS showed better performances than other algorithms. bAR, FS, and κ were between 70%-79%, 72%-79% and 0.38–0.58 respectively, and FDA, C5.0 and lsSVMrbf were the top three algorithms based on these three measures. MCC values were between 0.42–0.59, and FDA, C5.0 and SVMrbf outperformed other methods. Finally, the number of FN was between 8 and 19, and NSC, PLS and RLDA had the lowest false negative rates among others with 8 molecules. Conversely, RQDA had the highest false negative rate with 19 molecules. On the other hand, the number of FP obtained between 28 and 48, and lsSVMrbf, NN, C5.0 and FDA had the lowest false positive rates with 28, 30, 31 and 31 molecules, respectively. On the contrary, NB had the highest false positive rate with 48 molecules (FN and FP results not shown in the Table 1). These results showed that FDA from discriminant classifiers, C5.0 from tree-based classifiers and lsSVMrbf from kernel-based classifiers were the best performing algorithms among twenty-tree different statistical machine learning algorithms.

**Table 1:** Performance assessment of various statistical learning algorithms in virtual screening of compounds.

Classification Model	AR (%)	SE (%)	SP (%)	PPV (%)	NPV (%)	DR (%)	bAR (%)	FS (%)	MCC (%)	κ
**Discriminant Classifiers**										
** Linear discriminant analysis**	72.69	89.80	58.47	64.23	87.34	40.74	74.14	74.89	49.90	0.467
** Robust linear discriminant analysis**	75.93	**91.84**	62.71	67.16	**90.24**	**41.67**	77.27	77.59	55.96	0.529
** Quadratic discriminant analysis**	69.91	87.76	55.08	61.87	84.42	39.81	71.42	72.57	44.53	0.414
** Robust quadratic discriminant analysis**	73.61	80.61	67.80	67.52	80.81	36.57	74.20	73.49	48.37	0.476
** Mixture discriminant analysis**	75.93	90.82	63.56	67.42	89.29	41.20	77.19	77.39	55.53	0.528
** Flexible discriminant analysis**	**78.24**	89.80	**68.64**	**70.40**	89.01	40.74	**79.22**	**78.92**	**58.92**	**0.571**
** Nearest shrunken centroids**	74.07	**91.84**	59.32	65.22	**89.74**	**41.67**	75.58	76.27	53.03	0.494
**Decision Tree Classifiers**										
** Classification and regression trees**	72.22	88.78	58.47	63.97	86.25	40.28	73.63	74.36	48.71	0.457
**C5.0**	**78.24**	89.80	**68.64**	**70.40**	89.01	40.74	**79.22**	**78.92**	**58.92**	**0.571**
** J48**	77.31	89.80	66.95	69.29	88.76	40.74	78.37	78.22	57.40	0.554
** Conditional inference tree**	73.61	86.73	62.71	65.89	85.06	39.35	74.72	74.89	50.19	0.482
**Kernel-based Classifiers**										
** Support vector machines with linear kernel**	76.39	87.76	66.95	68.80	86.81	39.81	77.35	77.13	55.16	0.535
** Support vector machines with radial basis function kernel**	77.78	90.82	66.95	69.53	**89.77**	41.20	78.88	78.76	**58.53**	0.563
** Partial least squares**	74.07	**91.84**	59.32	65.22	**89.74**	**41.67**	75.58	76.27	53.03	0.494
** Least squares support vector machines with linear kernel**	73.15	90.82	58.47	64.49	88.46	41.20	74.65	75.42	51.09	0.476
** Least squares support vector machines with radial basis function kernel**	**78.70**	87.76	**71.19**	**71.67**	87.50	39.81	**79.47**	**78.90**	59.05	**0.578**
**Ensemble Classifiers**										
** Random forests**	76.85	88.78	66.95	69.05	87.78	40.28	77.86	77.68	56.27	0.544
** Bagged support vector machines**	76.39	88.78	66.10	68.50	87.64	40.28	77.44	77.33	55.51	0.535
** Bagged k-nearest neighbors**	75.46	90.82	62.71	66.92	89.16	41.20	76.76	77.06	54.79	0.520
**Other Classifiers**										
** Naïve bayes**	68.06	88.78	50.85	60.00	84.51	40.28	69.81	71.60	41.99	0.381
** Neural networks**	77.31	86.73	**69.49**	70.25	86.32	39.35	78.11	77.63	56.39	0.551
** k-Nearest neighbors**	76.85	90.82	65.25	68.46	89.53	41.20	78.04	78.07	57.03	0.546
** Learning vector quantization**	74.07	87.76	62.71	66.15	86.05	39.81	75.23	75.44	51.33	0.491

AR: Accuracy rate, SE: Sensitivity, SP: Specificity, PPV: Positive predictive value, NPV: Negative predictive value, DR: Detection rate, bAR: Balanced accuracy rate,

FS: F score, MCC: Matthews correlation coefficient, κ: Kappa statistic. Bold values indicate the top three winner algorithms in each performance measure

**Fig 1:**
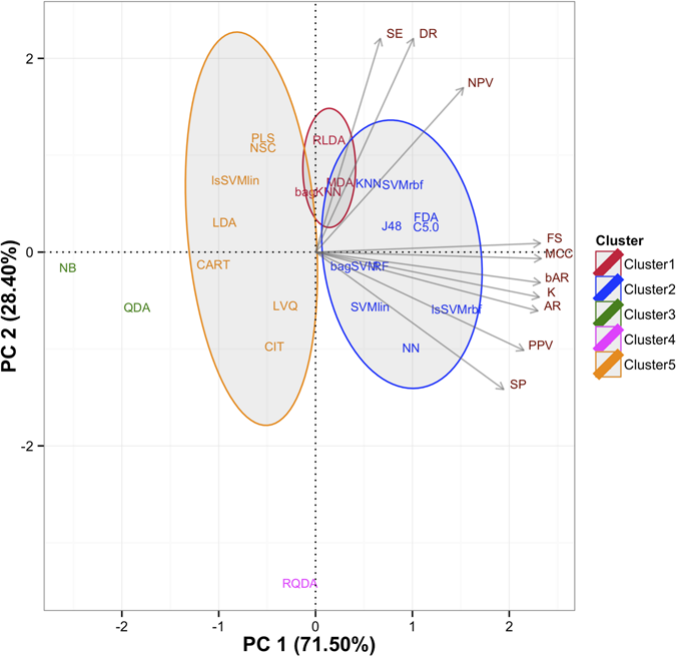
Principal component scores with loading biplot. Two principal components are explained almost all of the variability in the performance measures set. The first principal component accounted for 71.50% while the second principal component accounted for 28.40% of the variance of the performance measures data. Seven variables are loaded on the first principal component (AR: Accuracy rate, SP: Specificity, PPV: Positive predictive value, bAR: Balanced accuracy rate, FS: F score, MCC: Matthews correlation coefficient, κ: Kappa) whereas three variables (SE: Sensitivity, NPV: Negative predictive value, DR: Detection rate) are loaded on the second principal component.

Based on the PCA, there were two components and the first principal component (PC1) explained 71.50% of the variance while the second principal component (PC2) explained 28.40% of the variance. Hence, they explained almost all of the variability in the performance measures set. As can be seen from Fig 1, seven measures, AR, SP, PPV, bAR, FS, MCC and κ, loaded on the first component, whereas only three measures, SE, NPV and DR, loaded on the second component. Since the PC1 explains majority of the variability, we can crudely split the methods into two parts as the positive side of the PC1, including RLDA, MDA, bagKNN, KNN, SVMrbf, J48, FDA, C5.0, bagSVM, RF, SVMlin, NN and lsSVMrbf, and the negative side of the PC1, involving PLS, NSC, lsSVMlin, LDA, CART, LVQ, CIT, RQDA, QDA and NB. Moreover, the methods located in the positive side of the PC1 perform above average on all measures that loaded on PC1, which means they are performed well than the algorithms placed in the negative side of the PC1.

**Fig 2:**
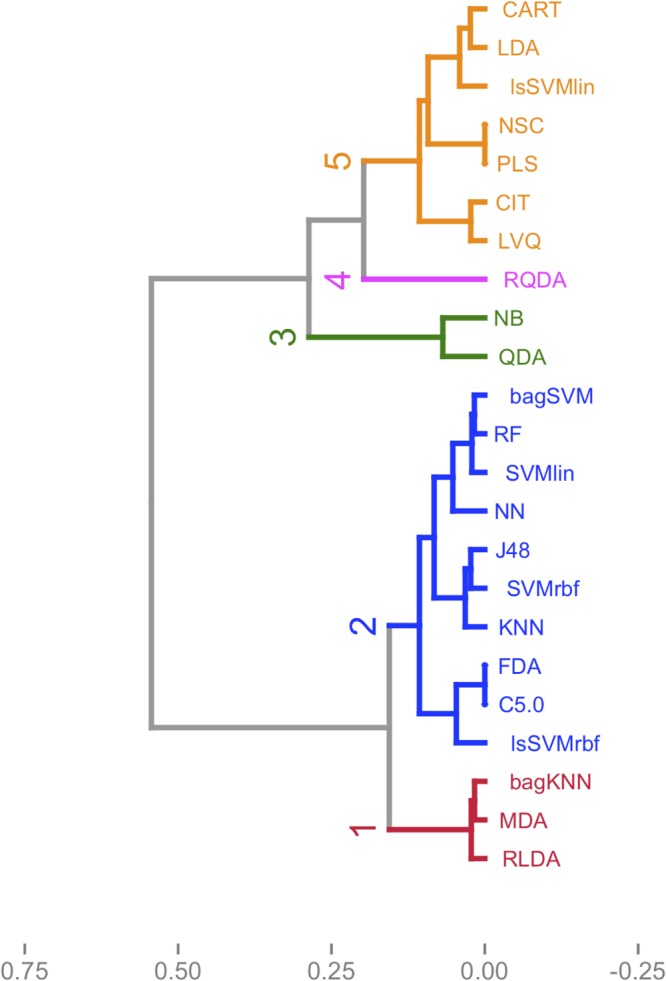
Hierarchical cluster dendrogram. The algorithms used in the study are clustered into five clusters. Cluster 1 and 2 involve the algorithms (RLDA: Robust linear discriminant analysis, bagKNN: Bagged k-nearest neighbors, MDA: Mixture discriminant analysis, KNN: k-Nearest neighbors, SVMrbf: Support vector machines with radial basis function kernel, FDA: Flexible discriminant analysis, J48, C5.0, NN: Neural networks, SVMlin: Support vector machines with linear kernel, lsSVMrbf: Least squares support vector machines with radial basis function kernel, RF: Random forests, bagSVM: Bagged support vector machines), which are loaded on the positive side of the first principal component, and cluster 3 to 5 include the algorithms (LDA: Linear discriminant analysis, lsSVMlin: Least squares support vector machines with linear kernel, NSC: Nearest shrunken centroids, PLS: Partial least squares, QDA: Quadratic discriminant analysis, RQDA: Robust quadratic discriminant analysis, CIT: Conditional inference tree, NB: Naïve bayes, LVQ: Learning vector quantization, CART: Classification and regression trees) that are loaded on the negative side of the first principal component.

We can also benefit from HC analysis results to support PC analysis results and to get more information about best performing algorithms. According to the dendrogram in Fig 2, which is derived by clustering analysis, the methods used in this study are clustered into five clusters. Cluster 1 and 2 contain the methods, which are fall into in the positive side of the PC1 whereas cluster 3 to 5 involve methods that are located in the negative side of the PC1. Therefore, we can conclude that cluster 1 and 2 include the best performing algorithms based on the results from the PC analysis. As seen from Fig 1, although the methods in the cluster 1 are taken part in the positive side of the both PCs, their loadings are very close to the origin of the PC1 and they are only explained by three performance measures, including SE, NPV and DR. On the other hand, the majority of the performance measures explain the methods in the cluster 2 and they are also situated in the positive side of the PC1. This means that the methods in the cluster 2 represent better performance than the methods in the cluster 1. Hence, we have determined to use the methods in the cluster 2 for our web-tool application: one discriminant classifier; FDA, two decision tree classifiers; C5.0 and J48, three kernel-based classifiers; lsSVMrbf, SVMrbf and SVMlin, two ensemble classifiers; RF and bagSVM, and two other classifiers; KNN and NN.

After selecting best-performed algorithms, to construct our web-tool, the training and the test sets, which are used for performance comparison of the methods in the *statistical machine learning methods* section, are combined in order to increase the sample size. Thus, we obtained a single training set, which contained 847 compounds (409 drug-like and 438 nondrug-like compounds). Then, we applied the same training procedure, as explained in the *model building* section, to this new training set. First, a z-score transformation is applied to center and scale the training set, then, tuning parameters are optimized using a 10 fold cross-validation with a 10 repeat.

The optimization results were as follows: for FDA, product degree and number of terms are acquired as 1 and 7, respectively, for C5.0, number of boosting iterations are selected as 10, a tree-based model is used whereas predictor winnowing is not used, for J48, confidence threshold is set as 0.25, for lsSVMrbf, optimal sigma parameter is obtained as 0.27, for SVMrbf, sigma and cost parameters are determined as 0.30 and 1, respectively, for SVMlin, cost parameter selected as 1, for RF, number of randomly selected predictors set as 2 and 500 trees are used, for bagSVM, number of bootstraps are set as 100 and radial basis function used as kernel, for NN, number of hidden units and weight decay are optimized as 19 and 0.1, respectively, and for KNN, number of neighbors are selected as 11. Finally, all best performing methods are fitted to the training set with the optimized value of the tuning parameters.

**Fig 3:**
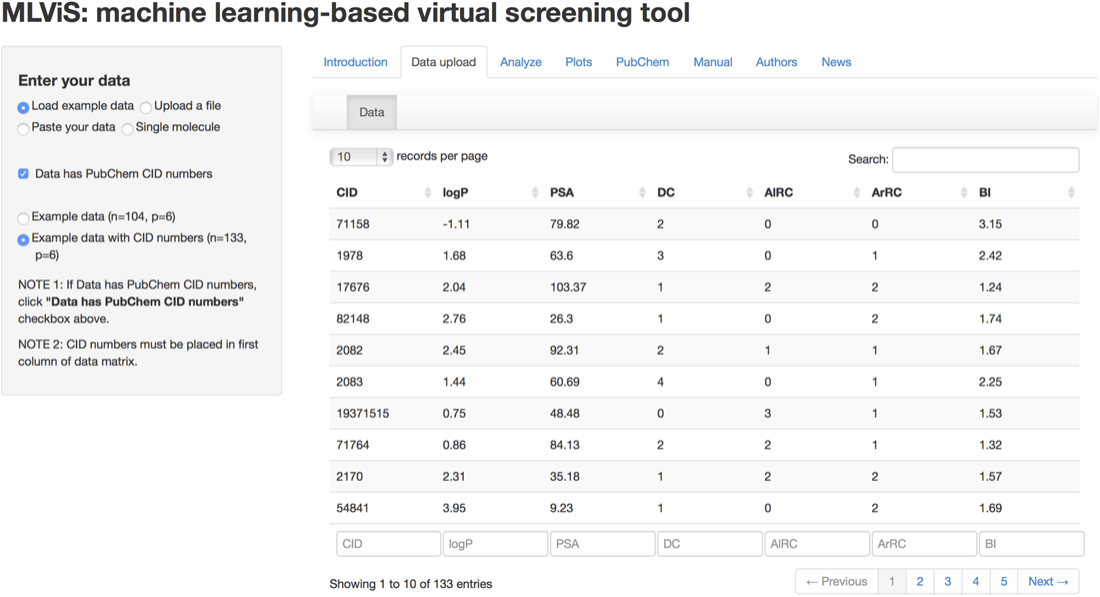
Data upload tab of the MLViS web-tool. Users can upload their files using upload file, paste data or single molecule options.

To build our web-tool, we have benefited from shiny package version 0.10.1 [26], which allows building interactive web applications with R software. The first step of using this web application is to upload a data file, which must have following six descriptors in precise order: logP, PSA DC, AlRC, ArRC and BI. As shown in Fig 3, users can either upload a file, which contain the data matrix, from their personal computers or directly paste their data set to the box in the tool.

**Fig 4:**
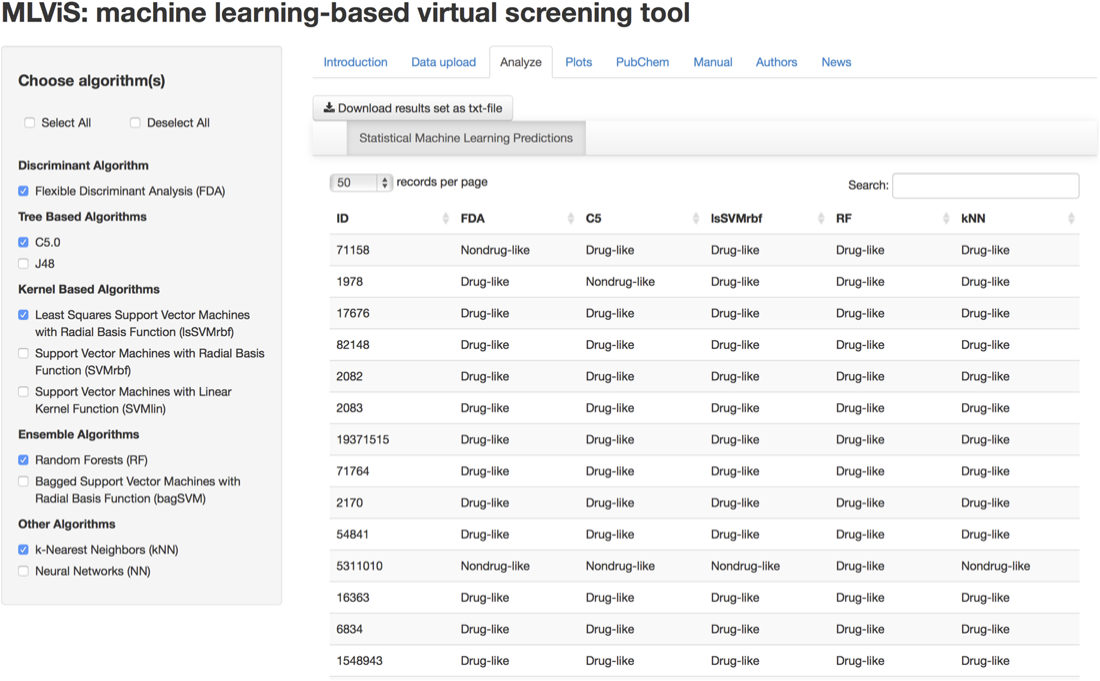
Analyze tab of the MLViS web-tool. A classification task can be performed using the statistical machine learning predictions.

If there is only one molecule, then the six descriptors of this molecule can be entered manually using single molecule option. There are two example data sets in the web-tool in order to help users to test the applicability of this application. After uploading the data set, one can move on to analyze this data set with these suggested statistical machine learning methods, as shown in Fig 4. As explained earlier, there are ten algorithms, which can classify compounds as drug-like and nondrug-like and users can select one of them, several of them or all of them at once. After selection of the method(s), the web-tool will be performed the analysis and showed the classification results immediately as drug-like or nondrug-like for each compound. Moreover, users can download the classification results to their personal computers by using download button in the page.

**Fig 5:**
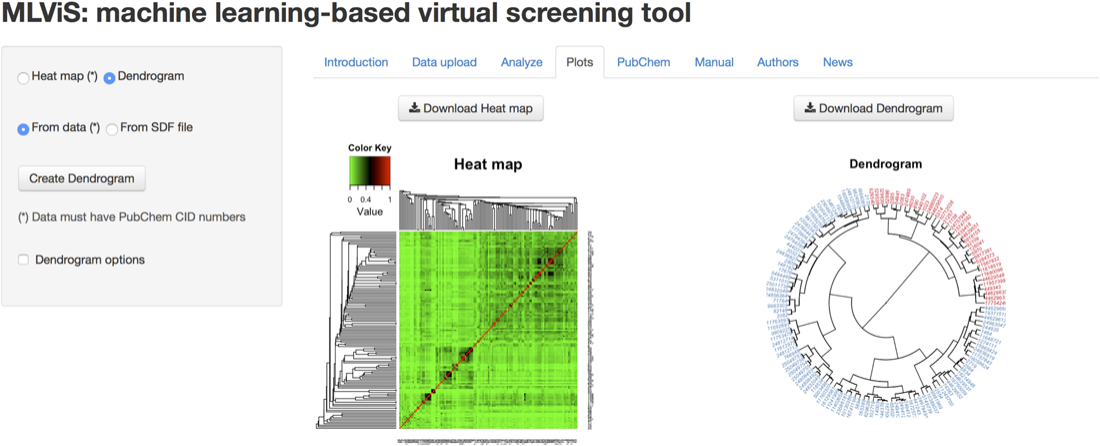
Plot tab of the MLViS web-tool. A dendrogram and a heat map can be created based on the compounds’ molecular similarity.

In the web-tool, hierarchical clustering analysis is used to cluster the compounds based on their similarity between molecular fingerprints and maximum common substructure search. To visualize the clustering results, a dendrogram and a heat map can be created, as shown in Fig 5, by using Rcpi package version 1.0.2 [23] and gplots package version 2.14.2 [27]. There are number of options for both dendrogram and heat map, such as method, metric, style, etc. For plotting, the data set must have compound identification (CID) number from PubChem. If the data have PubChem CID numbers, this must be placed in the first column of the data matrix. Alternatively, to create a dendrogram, users can upload an SDF file, which can be downloaded form PubChem database. There are also options in the web-tool for downloading both dendrogram and heat map as PDF file.

**Fig 6:**
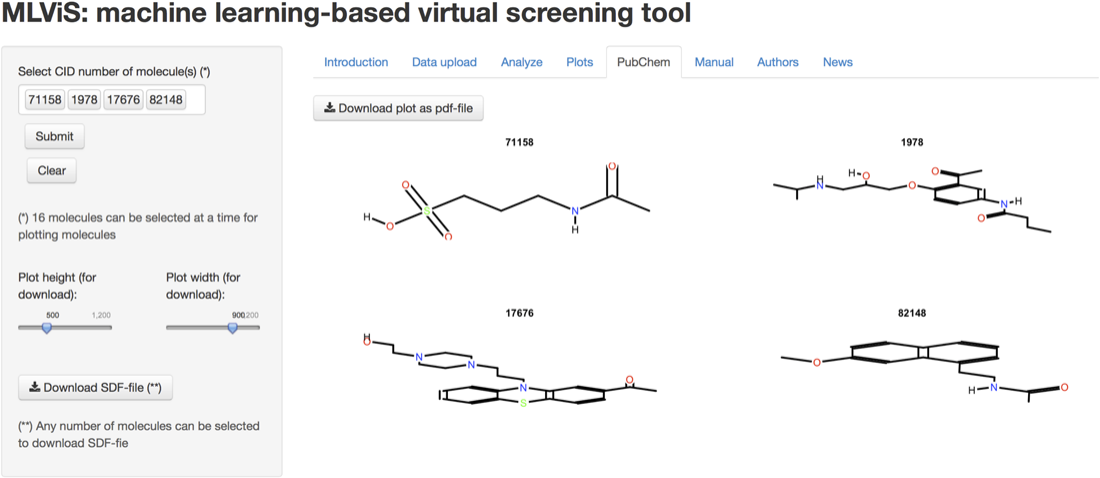
PubChem tab of the MLViS web-tool. Users can create and view molecular structures of compounds.

We have also benefited from ChemmineR package version 2.16.9 [28] to help users to plot two dimensional compound structure(s) of molecule(s) using PubChem CID number(s), as shown in Fig 6. Likewise, users can import compounds from PubChem and download the results as SDF file to their personal computers using CID numbers.

This application designed as a comprehensive machine learning-based virtual screening tool, which can perform both supervised (classification) and unsupervised (clustering) analysis. Although, machine learning algorithms have been extensively used in the early-phase of drug discovery studies, they are all diverse and applied on different data sets which makes them incomparable. Hence, we tested performances of numerous machine learning algorithms, which are widely used in the literature, on the same data set and selected best performing ones based on their performance measures. Rather than applying single classifiers, our work is more comprehensive than the other studies with testing the performance of twenty-three different statistical machine learning algorithms with different mathematical backgrounds for classification purpose. Providing the applicability of best performing algorithms in an easy-to-use web-tool is another originality of this study. Our classification performances were comparable with [5,7,12] and lower than [6,11]. We obtained 68–79% accuracy, while it was 70–78% in [5], 80–82% in [6], 70–75% in [7], 80–90% in [11] and 77–83% in [12]. Nevertheless, our models include only six descriptors as identified with various feature selection methods in [4], and these models are less complex as compared to classification models in [6,7,11,12]. However, we used the data from [4,5] where the number of compounds was 847 and relatively lower than the number of compounds (>5000) used in [6,7,11,12]. To our knowledge, there are two web tools available for screening small molecules. MolClass makes use of several machine learning algorithms and generates computational models from small molecule data sets using structural features identified in hit and non-hit molecules [38], and CHARMMing (Chemistry at Harvard Macromolecular Mechanics Interface and Graphics) performs quantitative structure activity relationship modeling using fifteen different machine learning algorithms [39]. Both tools benefit from the PubChem bioassay data sets. In practice, the number of molecules is significantly larger than that of active molecules. Although, the quite balanced data sets used in this study seem to be a shortage, it is well known that the machine learning algorithms, which are used in this study, require a balanced data set [40–42]. Therefore, before applying such algorithms, it is suggested to create a balanced data set from an imbalanced data set by using oversampling or undersampling methods, such as SMOTE [43], SMOTEBoost [44] and RUSBoost [45]. This web-tool includes best performing classification algorithms, plots derived from clustering methods and a link to the PubChem database, for researchers in the field. As a further research, we will improve the training performances by increasing the number of compounds and the number of features, and update MLViS based on the changes on optimal classification parameters.

### Availability and Future Directions

This application is freely available through http://www.biosoft.hacettepe.edu.tr/MLViS/ and it will be updated periodically upon the updated R packages, which are used in the tool, including Rcpi [23], caret [25], shiny [26], gplots [27] and ChemmineR [28].

## Supporting Information

S1 Table Training data set used in the study. This data set contains 631 compounds and six molecular descriptors.
(DOCX)

S2 Table Validation data set used in the study. This data set contains 216 compounds and six molecular descriptors.
(DOCX)
