# Supplementary material for: MLViS: A Web Tool for Machine Learning-Based Virtual Screening in Early-Phase of Drug Discovery and Development
Source: PLoS One. 2015 Apr 30;10(4):e0124600. doi: 10.1371/journal.pone.0124600 (PMC4415797; doi:10.1371/journal.pone.0124600)
Supplement: S1 Table — This data set contains 631 compounds and six molecular descriptors. (DOCX) [file pone.0124600.s001.docx]

**Table S1. Training data set used in the study.** This data set contains 631 compounds and six molecular descriptors.

| group | logP | PSA | DC | AlRC | ArRC | BI |
| --- | --- | --- | --- | --- | --- | --- |
| 1 | -6.82 | 309.14 | 14 | 4 | 0 | 1.35 |
| 1 | -0.44 | 125.64 | 2 | 0 | 1 | 2.18 |
| 1 | -2.73 | 113.52 | 4 | 3 | 0 | 1.65 |
| 1 | 1.18 | 94.83 | 3 | 4 | 0 | 1.7 |
| 1 | -0.8 | 74.69 | 2 | 2 | 0 | 1.66 |
| 1 | 2.44 | 43.07 | 0 | 1 | 3 | 1.59 |
| 1 | 2.84 | 29.46 | 2 | 0 | 1 | 2.22 |
| 1 | 2.31 | 26 | 1 | 3 | 0 | 1.79 |
| 1 | 0.43 | 47.89 | 4 | 1 | 0 | 2.36 |
| 1 | 0.05 | 37.3 | 2 | 0 | 0 | 2.75 |
| 1 | 7.91 | 53.03 | 0 | 0 | 3 | 1.54 |
| 1 | 4.93 | 13.59 | 0 | 1 | 2 | 1.48 |
| 1 | 1.7 | 61.83 | 2 | 1 | 1 | 2.21 |
| 1 | 3.99 | 46.71 | 2 | 1 | 2 | 1.37 |
| 1 | 1.76 | 26 | 1 | 0 | 1 | 2.13 |
| 1 | 2.12 | 111.87 | 3 | 1 | 2 | 1.63 |
| 1 | 0.86 | 78.29 | 0 | 1 | 1 | 1.9 |
| 1 | 3.12 | 54.05 | 2 | 2 | 2 | 1.45 |
| 1 | 3.91 | 93.59 | 2 | 2 | 2 | 1.49 |
| 1 | 0.7 | 122.84 | 5 | 2 | 1 | 1.7 |
| 1 | 3.41 | 53.48 | 1 | 2 | 2 | 0.96 |
| 1 | 4.46 | 40.64 | 1 | 2 | 3 | 1.01 |
| 1 | 0.46 | 46.53 | 3 | 0 | 1 | 1.98 |
| 1 | 6.27 | 54.37 | 1 | 2 | 2 | 1.18 |
| 1 | 1.83 | 60.12 | 1 | 2 | 1 | 1.33 |
| 1 | 1.71 | 74.6 | 2 | 0 | 0 | 2.92 |
| 1 | 3.94 | 48.48 | 0 | 2 | 2 | 1.45 |
| 1 | 1.33 | 37.3 | 2 | 0 | 1 | 2.36 |
| 1 | 4.28 | 22.82 | 0 | 2 | 2 | 1.36 |
| 1 | 1.14 | 94.83 | 3 | 4 | 0 | 1.71 |
| 1 | 2.53 | 103.61 | 2 | 0 | 2 | 1.96 |
| 1 | 3.56 | 142 | 2 | 2 | 2 | 1.46 |
| 1 | -1.77 | 136.1 | 2 | 1 | 1 | 2.16 |
| 1 | 3.57 | 30.66 | 1 | 1 | 1 | 1.82 |
| 1 | 3.83 | 72.51 | 2 | 6 | 1 | 1.42 |
| 1 | 3.6 | 17.07 | 1 | 0 | 1 | 2.46 |
| 1 | 1.17 | 90.34 | 0 | 4 | 0 | 1.08 |
| 1 | 2.28 | 80.35 | 2 | 2 | 2 | 1.47 |
| 1 | -2.04 | 61.82 | 0 | 2 | 0 | 2.23 |
| 1 | 2.78 | 118.81 | 2 | 1 | 4 | 1.17 |
| 1 | 0.64 | 106.76 | 2 | 1 | 0 | 2.57 |
| 1 | -2.06 | 26.3 | 1 | 0 | 0 | 3.21 |
| 1 | 3.62 | 59.29 | 1 | 1 | 2 | 1.68 |
| 1 | 3.42 | 99.88 | 3 | 1 | 1 | 2.18 |
| 1 | 2.86 | 48.19 | 1 | 1 | 2 | 1.58 |
| 1 | 2.8 | 39.89 | 1 | 0 | 1 | 2.25 |
| 1 | 3.24 | 26.48 | 0 | 1 | 1 | 1.72 |
| 1 | 3.88 | 52.48 | 0 | 1 | 2 | 1.47 |
| 1 | 1.61 | 26.3 | 1 | 1 | 1 | 1.97 |
| 1 | -0.5 | 90.13 | 3 | 1 | 0 | 1.85 |
| 1 | 0.4 | 71.2 | 2 | 2 | 2 | 1.42 |
| 1 | 3.61 | 58.35 | 2 | 1 | 2 | 1.31 |
| 1 | 3.54 | 46.61 | 0 | 1 | 2 | 1.59 |
| 1 | 1.66 | 193.26 | 4 | 3 | 0 | 2.13 |
| 1 | 3.32 | 35.53 | 0 | 0 | 1 | 2.39 |
| 1 | 7.25 | 22.82 | 0 | 0 | 3 | 1.37 |
| 1 | 3.61 | 27.18 | 0 | 1 | 2 | 1.73 |
| 1 | 2.67 | 75.25 | 1 | 1 | 2 | 1.79 |
| 1 | 1.88 | 12.36 | 2 | 1 | 1 | 1.86 |
| 1 | 5.23 | 17.82 | 0 | 1 | 3 | 1.55 |
| 1 | 2.12 | 39.54 | 1 | 2 | 2 | 1.48 |
| 1 | 1.08 | 52.28 | 1 | 4 | 1 | 1.5 |
| 1 | 4.68 | 13.59 | 0 | 2 | 2 | 1.59 |
| 1 | 0.14 | 159.82 | 5 | 3 | 2 | 1.48 |
| 1 | 1.08 | 0 | 0 | 0 | 0 | 3.13 |
| 1 | 2.75 | 13.59 | 1 | 1 | 2 | 1.57 |
| 1 | 4.01 | 20.23 | 1 | 4 | 0 | 1.62 |
| 1 | 1.14 | 94.83 | 3 | 4 | 0 | 1.71 |
| 1 | 3.04 | 22.82 | 0 | 3 | 1 | 1.51 |
| 1 | 2.92 | 32.67 | 0 | 1 | 2 | 1.63 |
| 1 | 1.4 | 54.88 | 1 | 1 | 1 | 2.13 |
| 1 | 4.22 | 52.78 | 1 | 1 | 1 | 1.72 |
| 1 | 3.75 | 37.3 | 2 | 0 | 2 | 1.74 |
| 1 | 3.66 | 136.36 | 2 | 2 | 2 | 1.33 |
| 1 | 5.91 | 40.46 | 2 | 0 | 2 | 1.96 |
| 1 | 5.48 | 40.46 | 2 | 0 | 2 | 1.96 |
| 1 | 1.71 | 137.23 | 3 | 5 | 3 | 0.92 |
| 1 | 2.84 | 94.73 | 0 | 1 | 2 | 1.66 |
| 1 | 3.16 | 22.82 | 0 | 0 | 2 | 1.68 |
| 1 | -2.82 | 172.2 | 4 | 4 | 0 | 1.47 |
| 1 | 3.59 | 60.69 | 4 | 0 | 2 | 1.38 |
| 1 | 1.82 | 107.86 | 2 | 0 | 2 | 1.5 |
| 1 | 1.81 | 85.87 | 2 | 3 | 2 | 0.94 |
| 1 | 0.92 | 40.46 | 3 | 0 | 1 | 2.07 |
| 1 | -0.43 | 113.28 | 2 | 1 | 1 | 2 |
| 1 | 1.38 | 106.95 | 1 | 3 | 2 | 1.09 |
| 1 | -0.47 | 180.05 | 6 | 3 | 2 | 1.45 |
| 1 | 2.6 | 66.8 | 1 | 2 | 2 | 1.12 |
| 1 | 4.05 | 37.47 | 1 | 0 | 3 | 1.39 |
| 1 | 0.37 | 20.23 | 1 | 0 | 1 | 2.5 |
| 1 | 2.13 | 94.26 | 2 | 1 | 1 | 1.84 |
| 1 | 1.94 | 25.95 | 1 | 2 | 2 | 1.51 |
| 1 | 0.55 | 60.69 | 4 | 0 | 1 | 2.18 |
| 1 | 1.71 | 137.23 | 3 | 5 | 3 | 0.92 |
| 1 | 1.14 | 204.26 | 5 | 3 | 0 | 2.13 |
| 1 | 4.23 | 40.46 | 2 | 3 | 1 | 1.36 |
| 1 | 3.63 | 37.3 | 1 | 3 | 1 | 1.36 |
| 1 | 3.3 | 63.6 | 1 | 0 | 1 | 2.42 |
| 1 | -0.08 | 20.23 | 1 | 0 | 0 | 1.63 |
| 1 | 4.25 | 40.46 | 2 | 3 | 1 | 1.58 |
| 1 | 0.9 | 89.41 | 1 | 1 | 1 | 1.69 |
| 1 | 1.04 | 160.83 | 3 | 5 | 2 | 1.19 |
| 1 | 3.62 | 52.6 | 1 | 1 | 1 | 2.21 |
| 1 | 5.33 | 52.6 | 0 | 0 | 2 | 1.63 |
| 1 | 3.93 | 33.9 | 0 | 1 | 2 | 1.29 |
| 1 | 5.64 | 91.35 | 3 | 1 | 3 | 1.2 |
| 1 | 4.62 | 35.53 | 2 | 1 | 1 | 1.78 |
| 1 | -1.95 | 81.65 | 1 | 2 | 1 | 1.71 |
| 1 | -0.81 | 64.07 | 2 | 1 | 0 | 2.09 |
| 1 | 4.65 | 9.23 | 1 | 0 | 2 | 1.66 |
| 1 | 1.96 | 57.53 | 2 | 4 | 0 | 1.63 |
| 1 | 3.12 | 86.3 | 1 | 2 | 2 | 1.22 |
| 1 | 3.78 | 46.26 | 0 | 1 | 2 | 1.71 |
| 1 | 3.76 | 37.3 | 1 | 0 | 2 | 1.77 |
| 1 | 8.93 | 76.74 | 2 | 3 | 1 | 1.02 |
| 1 | 1.39 | 37.3 | 2 | 1 | 0 | 2.48 |
| 1 | 0.77 | 80.43 | 2 | 2 | 2 | 1.63 |
| 1 | -0.77 | 84.58 | 3 | 2 | 0 | 2.02 |
| 1 | 3.35 | 46.53 | 1 | 0 | 1 | 2.21 |
| 1 | 4.68 | 85.89 | 3 | 1 | 2 | 1.22 |
| 1 | 1.75 | 48.48 | 1 | 3 | 1 | 1.32 |
| 1 | 1.09 | 71.2 | 2 | 2 | 2 | 1.36 |
| 1 | 3.56 | 24.72 | 2 | 0 | 1 | 2.26 |
| 1 | 1.15 | 25.95 | 2 | 1 | 0 | 2.08 |
| 1 | 3.98 | 50.89 | 1 | 1 | 2 | 1.32 |
| 1 | 0.52 | 94.83 | 3 | 4 | 0 | 1.45 |
| 1 | 0.87 | 60.12 | 1 | 4 | 1 | 1.59 |
| 1 | 3.64 | 37.3 | 1 | 0 | 1 | 2.14 |
| 1 | 3.19 | 77.76 | 3 | 2 | 0 | 1.72 |
| 1 | 2.88 | 82.92 | 2 | 3 | 2 | 0.9 |
| 1 | 2.99 | 27.18 | 0 | 1 | 2 | 1.48 |
| 1 | 2.91 | 114.67 | 4 | 3 | 2 | 1.2 |
| 1 | 3.62 | 68.53 | 1 | 0 | 3 | 1.56 |
| 1 | 3.77 | 87.13 | 1 | 2 | 3 | 1.17 |
| 1 | -0.82 | 41.99 | 2 | 1 | 0 | 2.24 |
| 1 | 1.43 | 60.69 | 4 | 0 | 1 | 2.15 |
| 1 | 1.52 | 125.4 | 0 | 4 | 3 | 0.7 |
| 1 | 2.88 | 17.07 | 1 | 1 | 1 | 1.92 |
| 1 | 1.8 | 89.76 | 0 | 3 | 2 | 0.98 |
| 1 | 3.22 | 54.37 | 1 | 0 | 2 | 1.7 |
| 1 | -0.79 | 89.65 | 2 | 2 | 0 | 1.76 |
| 1 | 3.15 | 38.67 | 2 | 1 | 1 | 1.76 |
| 1 | 1.49 | 87.08 | 1 | 2 | 1 | 1.33 |
| 1 | 4.4 | 86.99 | 3 | 1 | 1 | 1.51 |
| 1 | 3.91 | 33.82 | 1 | 3 | 1 | 1.38 |
| 1 | -0.63 | 47.73 | 1 | 1 | 0 | 2.52 |
| 1 | 5.03 | 74.68 | 1 | 2 | 2 | 1.2 |
| 1 | -1.76 | 77.76 | 4 | 0 | 1 | 2.33 |
| 1 | 3.29 | 33.82 | 1 | 3 | 1 | 1.56 |
| 1 | 2.1 | 30.66 | 1 | 0 | 1 | 2.26 |
| 1 | 3.83 | 32.3 | 0 | 1 | 0 | 2.76 |
| 1 | 1.93 | 60.04 | 2 | 2 | 2 | 1.31 |
| 1 | 5.46 | 64.48 | 1 | 1 | 3 | 1.36 |
| 1 | 3.47 | 49.66 | 2 | 1 | 2 | 1.59 |
| 1 | 2.35 | 90.86 | 2 | 2 | 2 | 1.19 |
| 1 | 4.54 | 72.83 | 1 | 3 | 0 | 1.52 |
| 1 | 2.55 | 48.77 | 0 | 2 | 2 | 1.49 |
| 1 | 1.24 | 105.26 | 4 | 1 | 1 | 1.99 |
| 1 | 5.06 | 12 | 1 | 2 | 2 | 1.36 |
| 1 | 0.35 | 103.7 | 5 | 0 | 0 | 5.22 |
| 1 | 2.73 | 46.18 | 1 | 2 | 2 | 1.42 |
| 1 | 2.66 | 12 | 1 | 2 | 0 | 2.16 |
| 1 | 3.5 | 54.37 | 1 | 4 | 0 | 1.49 |
| 1 | 1.26 | 42.09 | 2 | 0 | 2 | 1.79 |
| 1 | 3.52 | 26 | 1 | 3 | 0 | 1.63 |
| 1 | 2.87 | 88.76 | 0 | 2 | 2 | 1.26 |
| 1 | 4.59 | 30.66 | 0 | 0 | 2 | 2.33 |
| 1 | -0.71 | 112.85 | 1 | 1 | 0 | 2.51 |
| 1 | -0.61 | 149.5 | 5 | 2 | 1 | 1.29 |
| 1 | 0.83 | 38.69 | 2 | 0 | 1 | 2.44 |
| 1 | 2.08 | 26.3 | 1 | 1 | 1 | 2 |
| 1 | 0.88 | 55.82 | 2 | 2 | 2 | 1.29 |
| 1 | 2.18 | 39.89 | 2 | 0 | 1 | 2.42 |
| 1 | 5.02 | 27.05 | 0 | 1 | 2 | 1.4 |
| 1 | 3.22 | 30.18 | 0 | 2 | 2 | 1.55 |
| 1 | 3.79 | 50.89 | 1 | 4 | 1 | 1.46 |
| 1 | 1.15 | 67.89 | 1 | 2 | 0 | 1.73 |
| 1 | 0.22 | 75.49 | 3 | 2 | 0 | 1.8 |
| 1 | 3.57 | 83.83 | 2 | 1 | 0 | 2.22 |
| 1 | 0.76 | 63.28 | 2 | 4 | 1 | 1.59 |
| 1 | 0.8 | 71.14 | 1 | 2 | 0 | 2.15 |
| 1 | 0.57 | 80.35 | 2 | 4 | 1 | 1.4 |
| 1 | 0.7 | 80.35 | 2 | 5 | 1 | 1.36 |
| 1 | 1.39 | 71.9 | 2 | 1 | 2 | 1.47 |
| 1 | 1.88 | 76.23 | 0 | 2 | 2 | 1.09 |
| 1 | 6 | 113.49 | 4 | 2 | 2 | 1.36 |
| 1 | 3.4 | 112.01 | 1 | 1 | 2 | 1.62 |
| 1 | 1.13 | 26.48 | 0 | 2 | 0 | 1.74 |
| 1 | 1.84 | 98.42 | 1 | 1 | 1 | 2.25 |
| 1 | 2.53 | 107.65 | 1 | 1 | 1 | 2.26 |
| 1 | 2.92 | 98.42 | 1 | 1 | 1 | 2.23 |
| 1 | 2.26 | 98.42 | 1 | 1 | 1 | 2.34 |
| 1 | 3.21 | 37.3 | 1 | 4 | 0 | 1.58 |
| 1 | 0.21 | 71.84 | 2 | 2 | 1 | 1.68 |
| 1 | 3.78 | 37.3 | 1 | 4 | 0 | 1.54 |
| 1 | 4.69 | 12 | 1 | 1 | 2 | 1.57 |
| 1 | 4.12 | 158.05 | 5 | 1 | 3 | 1.31 |
| 1 | 0.26 | 94.66 | 1 | 3 | 1 | 1.38 |
| 1 | 0.7 | 67.78 | 1 | 2 | 2 | 1.54 |
| 1 | 3.42 | 60.12 | 1 | 1 | 2 | 1.53 |
| 1 | 2.59 | 149.13 | 3 | 2 | 1 | 1.54 |
| 1 | 0.2 | 96.31 | 1 | 2 | 1 | 1.41 |
| 1 | 0.19 | 48.48 | 0 | 3 | 1 | 1.48 |
| 1 | 3.59 | 22.82 | 0 | 0 | 2 | 1.94 |
| 1 | -1.24 | 206.6 | 8 | 6 | 0 | 1.16 |
| 1 | 4.15 | 46.53 | 1 | 4 | 0 | 1.64 |
| 1 | 3.04 | 32.59 | 2 | 1 | 1 | 1.82 |
| 1 | 2.96 | 209.26 | 4 | 4 | 3 | 1.23 |
| 1 | 3 | 49.81 | 0 | 1 | 2 | 1.42 |
| 1 | 3.59 | 27.69 | 1 | 2 | 2 | 1.18 |
| 1 | -2.89 | 100.1 | 4 | 3 | 0 | 1.61 |
| 1 | 3.65 | 54.68 | 1 | 2 | 2 | 1.58 |
| 1 | 6.2 | 12 | 1 | 3 | 0 | 1.46 |
| 1 | 2.82 | 86.3 | 1 | 2 | 2 | 1.3 |
| 1 | 1.67 | 46.18 | 2 | 1 | 2 | 1.42 |
| 1 | 0.52 | 40.46 | 3 | 0 | 1 | 2.29 |
| 1 | 2.22 | 46.17 | 2 | 1 | 2 | 1.76 |
| 1 | 0.89 | 53.48 | 1 | 2 | 1 | 1.59 |
| 1 | -0.99 | 105.59 | 2 | 5 | 0 | 1.59 |
| 1 | 0.25 | 44.12 | 0 | 2 | 0 | 1.76 |
| 1 | 5.36 | 49.73 | 1 | 2 | 3 | 1.03 |
| 1 | 1.53 | 45.25 | 3 | 0 | 2 | 1.6 |
| 1 | 2.34 | 93.59 | 1 | 2 | 1 | 1.12 |
| 1 | -0.77 | 24.1 | 2 | 1 | 0 | 2 |
| 1 | 0.58 | 77.45 | 1 | 3 | 1 | 1.31 |
| 1 | 1.89 | 106.3 | 2 | 2 | 1 | 1.67 |
| 1 | 0.76 | 37.66 | 2 | 2 | 0 | 1.79 |
| 1 | 2.24 | 124.29 | 4 | 2 | 0 | 1.96 |
| 1 | 0.59 | 101.63 | 1 | 2 | 2 | 1.27 |
| 1 | 1.02 | 94.83 | 3 | 4 | 0 | 1.45 |
| 1 | 2.1 | 17.07 | 2 | 0 | 1 | 2.28 |
| 1 | 1.65 | 30.66 | 2 | 0 | 1 | 2.01 |
| 1 | 2.18 | 39.89 | 1 | 0 | 1 | 2.01 |
| 1 | 3.53 | 66.07 | 0 | 2 | 2 | 1.21 |
| 1 | 4.16 | 33.82 | 1 | 2 | 1 | 1.43 |
| 1 | 3.52 | 34.14 | 0 | 4 | 0 | 1.54 |
| 1 | 3.26 | 52.48 | 0 | 1 | 2 | 1.51 |
| 1 | 3.36 | 52.48 | 0 | 1 | 2 | 1.6 |
| 1 | 3.2 | 46.53 | 2 | 0 | 2 | 1.57 |
| 1 | 3.03 | 29.46 | 2 | 0 | 2 | 1.54 |
| 1 | 1.39 | 20.23 | 2 | 0 | 1 | 2.33 |
| 1 | 3.35 | 25.78 | 2 | 1 | 1 | 2.01 |
| 1 | 1.74 | 94.3 | 1 | 2 | 2 | 1.35 |
| 1 | 2.83 | 55.94 | 1 | 3 | 1 | 1.38 |
| 1 | 5.19 | 108.59 | 2 | 1 | 4 | 1.08 |
| 1 | -0.49 | 177.6 | 4 | 1 | 2 | 1.36 |
| 1 | 2.45 | 26.3 | 1 | 2 | 1 | 1.47 |
| 1 | 0.47 | 86.17 | 2 | 0 | 1 | 1.92 |
| 1 | 3.07 | 27.69 | 1 | 1 | 2 | 1.59 |
| 1 | 2.92 | 112.34 | 1 | 4 | 2 | 1.17 |
| 1 | 2.46 | 74.51 | 0 | 3 | 2 | 0.96 |
| 1 | 3.94 | 169.58 | 4 | 2 | 2 | 1.31 |
| 1 | -0.39 | 60.09 | 1 | 1 | 2 | 1.36 |
| 1 | 2.25 | 30.66 | 1 | 1 | 1 | 1.62 |
| 1 | 1.52 | 107.18 | 1 | 2 | 1 | 1.13 |
| 1 | 4.18 | 69.92 | 4 | 0 | 2 | 1.27 |
| 1 | 3.83 | 114.99 | 5 | 3 | 2 | 1.16 |
| 1 | 0.79 | 72.65 | 1 | 3 | 1 | 1.34 |
| 1 | 5.14 | 12 | 1 | 1 | 2 | 1.54 |
| 1 | 0.12 | 140.85 | 1 | 3 | 1 | 1.46 |
| 1 | 0.89 | 62.75 | 3 | 0 | 1 | 2.26 |
| 1 | -0.34 | 81.55 | 3 | 3 | 1 | 1.59 |
| 1 | 3.41 | 85.74 | 0 | 5 | 0 | 1.37 |
| 1 | -1.35 | 78.87 | 2 | 2 | 0 | 1.71 |
| 1 | 1.36 | 68.55 | 2 | 0 | 2 | 1.85 |
| 1 | 0.54 | 42.52 | 2 | 0 | 1 | 2.18 |
| 1 | 1.16 | 82.41 | 2 | 1 | 1 | 1.9 |
| 1 | 0.66 | 71.9 | 2 | 0 | 2 | 1.87 |
| 1 | 2.56 | 12.89 | 1 | 2 | 1 | 1.66 |
| 1 | 3.27 | 188.71 | 3 | 4 | 0 | 1.71 |
| 1 | 7.1 | 22.82 | 0 | 0 | 3 | 1.5 |
| 1 | 1.72 | 192.55 | 1 | 5 | 0 | 1.34 |
| 1 | 0.27 | 97.72 | 1 | 3 | 1 | 1.27 |
| 1 | 1.42 | 60.69 | 4 | 0 | 1 | 2.38 |
| 1 | 7.1 | 54.05 | 2 | 1 | 3 | 1.12 |
| 1 | 3.68 | 43.37 | 0 | 4 | 0 | 1.6 |
| 1 | 3.6 | 37.3 | 1 | 4 | 0 | 1.35 |
| 1 | -2.01 | 72.68 | 1 | 2 | 0 | 1.87 |
| 1 | 4.9 | 77.78 | 0 | 2 | 2 | 1.3 |
| 1 | 2.64 | 107.37 | 1 | 1 | 2 | 1.35 |
| 1 | 2.93 | 41.83 | 0 | 1 | 2 | 1.27 |
| 1 | 6.62 | 101.94 | 2 | 2 | 2 | 1.35 |
| 1 | 2.48 | 85.21 | 2 | 1 | 1 | 1.7 |
| 1 | 2.18 | 59.59 | 2 | 0 | 1 | 2.33 |
| 1 | -0.18 | 97.9 | 1 | 3 | 0 | 1.84 |
| 1 | 2.43 | 43.05 | 1 | 1 | 1 | 1.87 |
| 1 | 3.47 | 94.26 | 2 | 3 | 0 | 1.58 |
| 1 | 1.36 | 26 | 1 | 1 | 1 | 1.48 |
| 1 | 4.57 | 96.22 | 3 | 1 | 1 | 1.65 |
| 1 | 0.97 | 66.48 | 0 | 2 | 2 | 0.98 |
| 1 | 3.06 | 43.07 | 0 | 1 | 3 | 1.55 |
| 1 | 3.83 | 66.07 | 0 | 2 | 2 | 1.35 |
| 1 | 4.52 | 33.82 | 1 | 2 | 1 | 1.63 |
| 1 | 4.69 | 37.14 | 0 | 3 | 2 | 1.33 |
| 1 | 1.32 | 53.47 | 2 | 1 | 1 | 1.85 |
| 1 | 2.04 | 53.47 | 3 | 1 | 2 | 1.5 |
| 1 | 1.84 | 40.07 | 0 | 1 | 1 | 1.65 |
| 1 | 4.01 | 26.48 | 0 | 2 | 1 | 1.35 |
| 1 | 3.6 | 110.16 | 2 | 2 | 2 | 1.08 |
| 1 | 3.42 | 90.97 | 2 | 3 | 4 | 1.14 |
| 1 | 2.74 | 37.3 | 1 | 0 | 0 | 3.5 |
| 1 | 1.33 | 141.67 | 1 | 3 | 1 | 1.4 |
| 1 | -4.29 | 284.55 | 14 | 2 | 2 | 1.59 |
| 1 | 2.83 | 43.05 | 1 | 1 | 1 | 2.03 |
| 1 | 4.7 | 74.3 | 0 | 0 | 2 | 1.78 |
| 1 | 2.79 | 185.15 | 3 | 6 | 3 | 1.09 |
| 1 | 2.59 | 191.87 | 3 | 6 | 3 | 1.09 |
| 1 | 2.96 | 63.6 | 1 | 1 | 2 | 1.71 |
| 1 | -0.76 | 115.08 | 3 | 2 | 0 | 1.78 |
| 1 | 2.68 | 81.91 | 1 | 3 | 2 | 1 |
| 1 | 1.21 | 56.61 | 0 | 2 | 1 | 1.66 |
| 0 | -1.23 | 146.04 | 6 | 0 | 0 | 4.88 |
| 0 | 0.06 | 118.05 | 3 | 3 | 1 | 1.29 |
| 0 | -5.28 | 225.94 | 7 | 3 | 0 | 1.5 |
| 0 | 3.82 | 215.01 | 4 | 4 | 1 | 1.34 |
| 0 | 0.9 | 169.15 | 11 | 0 | 1 | 3.24 |
| 0 | -0.29 | 318 | 6 | 6 | 0 | 1.01 |
| 0 | -4.13 | 189.53 | 8 | 2 | 0 | 2.18 |
| 0 | -7.06 | 282.54 | 7 | 3 | 0 | 1.67 |
| 0 | -7.06 | 282.54 | 7 | 3 | 0 | 1.67 |
| 0 | 1.27 | 139.46 | 2 | 0 | 1 | 2.22 |
| 0 | 2.05 | 108.74 | 6 | 0 | 0 | 5.82 |
| 0 | 2.62 | 88.51 | 5 | 0 | 0 | 5.63 |
| 0 | -0.88 | 313.24 | 2 | 10 | 0 | 0.73 |
| 0 | -2.13 | 107.22 | 5 | 1 | 0 | 2.48 |
| 0 | -1.26 | 110.24 | 7 | 0 | 0 | 4.47 |
| 0 | 0.29 | 71.44 | 6 | 0 | 1 | 2.6 |
| 0 | -0.65 | 96.74 | 6 | 0 | 0 | 4.72 |
| 0 | -2.61 | 100.12 | 7 | 1 | 0 | 2.58 |
| 0 | -0.29 | 71.44 | 6 | 0 | 0 | 4.84 |
| 0 | -2.04 | 71.44 | 6 | 0 | 0 | 4.02 |
| 0 | -0.18 | 71.44 | 6 | 0 | 1 | 2.71 |
| 0 | -1.29 | 85.03 | 5 | 1 | 0 | 2.57 |
| 0 | -2.64 | 91.67 | 7 | 0 | 0 | 4.47 |
| 0 | -2.18 | 91.67 | 7 | 0 | 0 | 4.68 |
| 0 | -0.47 | 91.67 | 7 | 0 | 1 | 2.65 |
| 0 | -3.12 | 88.51 | 7 | 0 | 0 | 4.78 |
| 0 | -2.76 | 88.51 | 7 | 0 | 0 | 4.84 |
| 0 | -0.58 | 71.44 | 6 | 0 | 0 | 4.62 |
| 0 | -0.02 | 71.44 | 6 | 0 | 0 | 4.72 |
| 0 | -0.08 | 71.44 | 6 | 0 | 0 | 4.78 |
| 0 | 0.9 | 169.15 | 11 | 0 | 1 | 3.24 |
| 0 | 0.94 | 175.24 | 7 | 2 | 1 | 1.41 |
| 0 | -2.13 | 107.22 | 5 | 1 | 0 | 2.48 |
| 0 | -3.82 | 187.77 | 11 | 0 | 0 | 4.84 |
| 0 | 4.3 | 63.24 | 3 | 0 | 2 | 1.98 |
| 0 | 0.31 | 113.28 | 2 | 0 | 2 | 1.71 |
| 0 | -0.14 | 136.1 | 1 | 1 | 2 | 1.66 |
| 0 | -0.21 | 122.51 | 2 | 0 | 2 | 1.62 |
| 0 | -0.28 | 136.1 | 1 | 1 | 2 | 1.66 |
| 0 | -2.13 | 136.1 | 2 | 1 | 1 | 2.01 |
| 0 | -1.04 | 156.33 | 2 | 1 | 2 | 1.62 |
| 0 | -2.26 | 175.34 | 2 | 1 | 2 | 1.75 |
| 0 | -1.06 | 141.52 | 2 | 0 | 2 | 1.64 |
| 0 | -7.29 | 290.4 | 9 | 1 | 0 | 3.16 |
| 0 | 2.32 | 150.92 | 5 | 1 | 1 | 2.39 |
| 0 | -6.71 | 273.31 | 8 | 3 | 0 | 1.52 |
| 0 | -5.54 | 245.87 | 9 | 2 | 0 | 1.99 |
| 0 | -3.04 | 357.59 | 13 | 1 | 1 | 2.61 |
| 0 | -0.19 | 74.43 | 3 | 2 | 0 | 1.89 |
| 0 | -0.01 | 127.3 | 3 | 3 | 1 | 1.17 |
| 0 | 1.13 | 84.78 | 3 | 3 | 2 | 1.21 |
| 0 | 0.45 | 142.57 | 3 | 0 | 2 | 2.07 |
| 0 | 4.21 | 114.92 | 4 | 2 | 4 | 1 |
| 0 | 0.94 | 175.24 | 7 | 2 | 1 | 1.41 |
| 0 | 2.78 | 120.56 | 5 | 1 | 2 | 1.43 |
| 0 | -0.43 | 113.28 | 2 | 1 | 1 | 2 |
| 0 | 0.46 | 190.92 | 7 | 5 | 0 | 1.12 |
| 0 | 1.85 | 156.53 | 4 | 0 | 2 | 1.92 |
| 0 | -1.88 | 90.23 | 4 | 2 | 0 | 1.79 |
| 0 | 6.92 | 84.88 | 1 | 2 | 4 | 0.99 |
| 0 | 5.27 | 100.9 | 6 | 2 | 3 | 1.22 |
| 0 | 3.39 | 74.6 | 3 | 1 | 2 | 1.27 |
| 0 | -5.75 | 246.17 | 7 | 3 | 0 | 1.5 |
| 0 | 4.98 | 60.69 | 3 | 3 | 0 | 1.56 |
| 0 | -4.52 | 189.83 | 6 | 3 | 0 | 1.66 |
| 0 | -1.03 | 168.44 | 6 | 2 | 1 | 1.42 |
| 0 | 4.01 | 161.7 | 6 | 2 | 2 | 1.49 |
| 0 | -0.82 | 111.39 | 1 | 0 | 1 | 2.29 |
| 0 | 4.78 | 84.71 | 4 | 1 | 4 | 1.37 |
| 0 | -4.77 | 211.78 | 5 | 2 | 0 | 1.78 |
| 0 | -1.06 | 89.87 | 3 | 3 | 0 | 1.73 |
| 0 | -2.57 | 130.47 | 5 | 2 | 0 | 2.04 |
| 0 | -3.14 | 276.01 | 12 | 1 | 2 | 1.79 |
| 0 | -4.26 | 239.04 | 11 | 1 | 1 | 2.24 |
| 0 | 4.43 | 12.89 | 1 | 3 | 1 | 1.29 |
| 0 | -2.49 | 125.77 | 5 | 1 | 1 | 2.19 |
| 0 | 4.09 | 51.21 | 4 | 0 | 1 | 2.7 |
| 0 | 2.95 | 51.21 | 3 | 0 | 1 | 2.46 |
| 0 | 2.49 | 153.3 | 6 | 1 | 1 | 2.13 |
| 0 | 3.1 | 131.56 | 6 | 2 | 1 | 1.79 |
| 0 | 2.63 | 159.24 | 5 | 1 | 1 | 2.09 |
| 0 | 0.76 | 177.01 | 2 | 4 | 1 | 1.11 |
| 0 | -2.97 | 200.41 | 8 | 0 | 0 | 5.51 |
| 0 | -5.04 | 247.89 | 5 | 2 | 0 | 1.69 |
| 0 | 0.82 | 93.41 | 1 | 1 | 1 | 1.94 |
| 0 | -1.89 | 105.45 | 4 | 1 | 0 | 2.68 |
| 0 | -0.79 | 279.12 | 13 | 1 | 0 | 3.4 |
| 0 | -0.66 | 206.48 | 7 | 0 | 1 | 2.93 |
| 0 | -1.9 | 163.11 | 10 | 0 | 1 | 2.86 |
| 0 | 6.76 | 54.37 | 1 | 1 | 2 | 1.44 |
| 0 | 1.32 | 125.26 | 3 | 3 | 1 | 1.44 |
| 0 | -1.52 | 251.62 | 12 | 0 | 1 | 2.96 |
| 0 | -2.98 | 113.52 | 3 | 3 | 0 | 1.71 |
| 0 | -2.29 | 133.69 | 5 | 3 | 0 | 1.65 |
| 0 | 3.64 | 54.37 | 2 | 0 | 2 | 1.52 |
| 0 | 4.96 | 96.89 | 3 | 0 | 3 | 1.63 |
| 0 | -6.24 | 297.04 | 10 | 3 | 0 | 1.86 |
| 0 | -4.3 | 174.56 | 6 | 0 | 0 | 4.04 |
| 0 | -3.71 | 169.3 | 7 | 2 | 0 | 1.9 |
| 0 | -1.52 | 275.02 | 9 | 1 | 1 | 1.85 |
| 0 | -2.26 | 361.86 | 17 | 0 | 2 | 2.45 |
| 0 | 1.39 | 171.56 | 9 | 2 | 1 | 1.69 |
| 0 | 2.49 | 153.3 | 6 | 1 | 1 | 2.13 |
| 0 | -0.86 | 241.33 | 14 | 1 | 2 | 1.76 |
| 0 | -4.31 | 189.53 | 8 | 2 | 0 | 2.11 |
| 0 | -3.58 | 275.66 | 7 | 1 | 0 | 2.78 |
| 0 | 0.72 | 121.1 | 7 | 0 | 2 | 1.96 |
| 0 | 2.73 | 114.81 | 6 | 0 | 2 | 2.22 |
| 0 | 0.99 | 114.81 | 6 | 0 | 1 | 2.75 |
| 0 | -1.1 | 411.25 | 16 | 1 | 2 | 1.88 |
| 0 | 0.35 | 295.87 | 12 | 2 | 3 | 1.24 |
| 0 | 2.84 | 194.09 | 11 | 0 | 2 | 2.26 |
| 0 | 4.33 | 227.91 | 11 | 1 | 3 | 1.48 |
| 0 | -5.4 | 226.2 | 6 | 3 | 0 | 1.54 |
| 0 | 7.02 | 37.3 | 1 | 0 | 0 | 2.92 |
| 0 | 7.02 | 37.3 | 1 | 0 | 0 | 2.92 |
| 0 | 6.57 | 84.03 | 1 | 0 | 0 | 3.67 |
| 0 | -1.36 | 107.58 | 3 | 2 | 0 | 2.09 |
| 0 | 5.32 | 112.84 | 2 | 3 | 2 | 1.12 |
| 0 | 5.07 | 109.35 | 4 | 1 | 3 | 1.42 |
| 0 | 3.02 | 174.82 | 4 | 2 | 2 | 1.27 |
| 0 | 3.04 | 161.7 | 6 | 2 | 2 | 1.46 |
| 0 | 4.07 | 141.47 | 5 | 2 | 2 | 1.45 |
| 0 | 3.04 | 161.7 | 6 | 2 | 2 | 1.46 |
| 0 | 8.27 | 84.71 | 2 | 1 | 6 | 1.07 |
| 0 | 3.83 | 114.99 | 5 | 3 | 2 | 1.16 |
| 0 | -1.39 | 200.19 | 7 | 2 | 1 | 1.64 |
| 0 | 2.46 | 64.52 | 2 | 0 | 3 | 1.36 |
| 0 | -1.14 | 337.28 | 13 | 3 | 2 | 0.98 |
| 0 | 2.24 | 114.81 | 7 | 0 | 3 | 1.69 |
| 0 | 5.44 | 36.92 | 0 | 0 | 1 | 1.99 |
| 0 | -2.22 | 256.75 | 9 | 1 | 1 | 2.19 |
| 0 | 5.19 | 156.38 | 6 | 3 | 2 | 1.2 |
| 0 | -3.66 | 319.57 | 10 | 3 | 1 | 1.44 |
| 0 | 2.03 | 221.27 | 8 | 2 | 2 | 1.46 |
| 0 | -1.51 | 71.44 | 6 | 0 | 0 | 4.28 |
| 0 | -2.04 | 108.74 | 7 | 0 | 0 | 4.84 |
| 0 | -0.3 | 87.23 | 7 | 0 | 2 | 1.97 |
| 0 | -2.35 | 199.67 | 6 | 2 | 0 | 1.96 |
| 0 | -6.98 | 302.51 | 8 | 3 | 0 | 1.49 |
| 0 | 2.06 | 141.29 | 5 | 0 | 3 | 1.48 |
| 0 | -6.7 | 294.07 | 7 | 5 | 0 | 1.03 |
| 0 | -5.85 | 234.06 | 8 | 1 | 0 | 2.96 |
| 0 | 2.9 | 234.86 | 10 | 1 | 5 | 1.16 |
| 0 | 1.6 | 155.51 | 9 | 1 | 2 | 1.58 |
| 0 | 0.8 | 146.04 | 9 | 0 | 2 | 2.09 |
| 0 | -1.86 | 267.95 | 10 | 0 | 3 | 1.69 |
| 0 | 1.56 | 183.65 | 4 | 3 | 1 | 1.31 |
| 0 | 4.5 | 37.3 | 1 | 4 | 0 | 1.35 |
| 0 | 4.27 | 108.74 | 5 | 0 | 2 | 2.03 |
| 0 | 0.04 | 164.05 | 3 | 0 | 1 | 2.29 |
| 0 | 5.39 | 92.68 | 3 | 0 | 3 | 1.35 |
| 0 | 4.14 | 95.03 | 2 | 0 | 0 | 3.36 |
| 0 | -3 | 174.56 | 7 | 0 | 1 | 2.3 |
| 0 | -1.5 | 256.75 | 9 | 0 | 1 | 3.07 |
| 0 | -6.66 | 310.39 | 8 | 5 | 0 | 0.97 |
| 0 | 4 | 114.81 | 6 | 0 | 3 | 1.68 |
| 0 | 4.01 | 114.81 | 6 | 0 | 3 | 1.68 |
| 0 | 3.65 | 80.81 | 3 | 1 | 2 | 1.27 |
| 0 | 0.64 | 271.85 | 12 | 0 | 1 | 3.15 |
| 0 | -3.36 | 134.1 | 4 | 0 | 0 | 3.81 |
| 0 | -3.78 | 166.72 | 6 | 1 | 0 | 2.54 |
| 0 | -6.63 | 282.54 | 7 | 3 | 0 | 1.52 |
| 0 | -1.97 | 164.92 | 6 | 2 | 1 | 1.64 |
| 0 | -1.14 | 110.55 | 4 | 2 | 1 | 1.58 |
| 0 | -1.31 | 149.66 | 10 | 0 | 1 | 2.94 |
| 0 | 0.22 | 156.47 | 6 | 1 | 0 | 3 |
| 0 | 1.02 | 127.78 | 4 | 0 | 2 | 1.75 |
| 0 | -3.85 | 146.49 | 6 | 1 | 0 | 2.54 |
| 0 | -6.45 | 425.87 | 20 | 2 | 2 | 1.42 |
| 0 | 3.32 | 119.68 | 2 | 2 | 2 | 1.22 |
| 0 | 4.38 | 130.56 | 4 | 1 | 3 | 1.32 |
| 0 | 4.58 | 89.9 | 5 | 1 | 2 | 1.68 |
| 0 | 5.58 | 4.93 | 2 | 0 | 4 | 1.4 |
| 0 | 5.45 | 20.23 | 1 | 0 | 0 | 2.78 |
| 0 | 0.46 | 104.06 | 3 | 1 | 0 | 2.53 |
| 0 | 1.03 | 189.41 | 8 | 0 | 3 | 1.65 |
| 0 | -2.95 | 140.92 | 4 | 2 | 0 | 1.86 |
| 0 | 2.15 | 46.61 | 4 | 2 | 2 | 1.25 |
| 0 | 6.16 | 37.3 | 1 | 0 | 1 | 2.51 |
| 0 | -2.73 | 122.23 | 6 | 2 | 1 | 1.65 |
| 0 | -3.67 | 196.22 | 5 | 1 | 2 | 1.49 |
| 0 | -1.78 | 157.57 | 4 | 2 | 0 | 1.47 |
| 0 | -6.91 | 311.16 | 9 | 3 | 0 | 1.47 |
| 0 | -5.38 | 232.01 | 6 | 2 | 0 | 1.78 |
| 0 | -5.64 | 271.85 | 15 | 0 | 2 | 2.07 |
| 0 | 1.89 | 137.35 | 3 | 1 | 2 | 1.9 |
| 0 | -3.08 | 133.49 | 5 | 3 | 0 | 1.59 |
| 0 | 4.97 | 122.65 | 7 | 0 | 4 | 1.32 |
| 0 | 5.78 | 77.76 | 3 | 4 | 0 | 1.46 |
| 0 | 2.87 | 62.75 | 2 | 0 | 3 | 1.49 |
| 0 | -4.77 | 188.18 | 5 | 3 | 0 | 1.58 |
| 0 | 2.16 | 108.94 | 1 | 0 | 0 | 4.57 |
| 0 | 3.09 | 151.76 | 1 | 0 | 0 | 4.54 |
| 0 | 3.1 | 77.85 | 1 | 2 | 2 | 1.41 |
| 0 | 4.34 | 126.64 | 3 | 1 | 4 | 1.26 |
| 0 | -3.04 | 128.65 | 5 | 2 | 0 | 2.01 |
| 0 | -4.78 | 186.35 | 5 | 2 | 0 | 1.81 |
| 0 | -0.65 | 115.45 | 4 | 1 | 0 | 2.47 |
| 0 | 4.96 | 57.19 | 3 | 5 | 3 | 0.74 |
| 0 | -4.36 | 155.89 | 5 | 3 | 0 | 1.5 |
| 0 | 4.82 | 218.23 | 4 | 3 | 4 | 1.05 |
| 0 | 4.31 | 151.47 | 3 | 3 | 3 | 1.06 |
| 0 | 2.71 | 123.83 | 3 | 1 | 2 | 1.37 |
| 0 | -4.31 | 189.53 | 8 | 2 | 0 | 2.11 |
| 0 | -2.86 | 174.58 | 6 | 0 | 1 | 2.51 |
| 0 | -4.52 | 189.83 | 6 | 3 | 0 | 1.53 |
| 0 | -0.86 | 71.44 | 6 | 0 | 0 | 4.68 |
| 0 | -1.85 | 248.14 | 10 | 1 | 1 | 2.26 |
| 0 | -4.23 | 147.54 | 6 | 0 | 0 | 4.07 |
| 0 | -2.13 | 107.22 | 5 | 1 | 0 | 2.48 |
| 0 | 4.54 | 100.9 | 4 | 1 | 3 | 1.16 |
| 0 | -5.03 | 317.53 | 8 | 3 | 0 | 1.49 |
| 0 | -6.13 | 294.67 | 10 | 3 | 0 | 1.77 |
| 0 | -7.06 | 293.28 | 9 | 3 | 0 | 1.49 |
| 0 | 1.41 | 75.51 | 0 | 2 | 2 | 1.15 |
| 0 | 1.34 | 117.52 | 3 | 1 | 3 | 1.27 |
| 0 | 2.82 | 66.7 | 5 | 1 | 3 | 1.21 |
| 0 | -6.78 | 254.93 | 11 | 2 | 0 | 2.2 |
| 0 | 0.46 | 79.67 | 3 | 2 | 0 | 1.47 |
| 0 | -4.28 | 86.71 | 3 | 2 | 0 | 2.04 |
| 0 | -6.48 | 285.12 | 8 | 4 | 0 | 1.14 |
| 0 | 2.92 | 74.03 | 3 | 2 | 1 | 1.38 |
| 0 | -1.26 | 176.03 | 4 | 2 | 0 | 1.74 |
| 0 | -0.27 | 156.47 | 9 | 2 | 2 | 1.58 |
| 0 | -3.55 | 155.44 | 4 | 2 | 0 | 1.86 |
| 0 | -3.32 | 155.44 | 4 | 2 | 0 | 1.82 |
| 0 | -9.36 | 406.75 | 9 | 5 | 0 | 1.2 |
| 0 | -4.17 | 169.86 | 5 | 3 | 0 | 1.58 |
| 0 | -3.33 | 133.49 | 4 | 3 | 0 | 1.65 |
| 0 | -4.31 | 189.53 | 8 | 2 | 0 | 2.11 |
| 0 | -5.28 | 243.9 | 10 | 2 | 0 | 2.4 |
| 0 | -2.3 | 188.92 | 4 | 2 | 0 | 1.74 |
| 0 | -1.48 | 130.91 | 4 | 2 | 0 | 1.61 |
| 0 | -0.84 | 231.39 | 13 | 0 | 1 | 3.52 |
| 0 | -4.93 | 317.75 | 10 | 4 | 0 | 1.32 |
| 0 | -8.95 | 346.74 | 10 | 1 | 0 | 3.42 |
| 0 | -9.81 | 376.2 | 11 | 1 | 0 | 3.26 |
| 0 | -4.58 | 175.67 | 5 | 2 | 0 | 1.98 |
| 0 | -3.55 | 155.44 | 4 | 2 | 0 | 1.97 |
| 0 | -3.4 | 155.44 | 4 | 2 | 0 | 1.98 |
| 0 | 3.08 | 37.3 | 1 | 2 | 2 | 1.36 |
| 0 | -6.68 | 265.47 | 7 | 3 | 0 | 1.52 |
| 0 | 1.03 | 74.6 | 2 | 0 | 1 | 2.19 |
| 0 | 1.49 | 134.36 | 4 | 1 | 0 | 2.82 |
| 0 | -1.95 | 98.74 | 5 | 2 | 1 | 1.57 |
| 0 | -4.99 | 247.97 | 7 | 1 | 0 | 2.97 |
| 0 | -4.99 | 247.97 | 7 | 1 | 0 | 2.97 |
| 0 | -3.28 | 194.6 | 7 | 2 | 0 | 2.03 |
| 0 | -8.45 | 388.29 | 17 | 5 | 0 | 1.1 |
| 0 | -8.78 | 388.29 | 17 | 5 | 0 | 1.1 |
| 0 | -7.98 | 408.3 | 10 | 6 | 0 | 0.94 |
| 0 | 0.99 | 119.84 | 1 | 0 | 3 | 1.51 |
| 0 | -2.6 | 148.02 | 5 | 2 | 0 | 1.74 |
| 0 | 2.02 | 55.12 | 2 | 1 | 1 | 1.64 |
| 0 | -2.75 | 203.44 | 7 | 2 | 0 | 2.07 |
| 0 | -0.61 | 107.33 | 5 | 2 | 1 | 1.55 |
| 0 | 0.75 | 124.9 | 4 | 2 | 2 | 1.53 |
| 0 | 1.31 | 156.53 | 3 | 0 | 1 | 2 |
| 0 | 1.2 | 136.78 | 4 | 1 | 2 | 1.69 |
| 0 | 4.56 | 67.39 | 4 | 0 | 3 | 1.37 |
| 0 | 5.49 | 142.87 | 3 | 3 | 1 | 1.51 |
| 0 | 2.98 | 135.04 | 7 | 0 | 3 | 1.69 |
| 0 | 2.98 | 135.04 | 7 | 0 | 3 | 1.69 |
| 0 | -1.61 | 53.09 | 3 | 0 | 2 | 1.76 |
| 0 | 0.83 | 139.72 | 12 | 0 | 1 | 3.1 |
| 0 | 6.46 | 94.8 | 1 | 1 | 4 | 1 |
| 0 | -1.07 | 305.1 | 11 | 3 | 0 | 1.7 |
| 0 | 2.18 | 110.61 | 2 | 4 | 2 | 0.88 |
| 0 | -3.54 | 158.3 | 6 | 2 | 0 | 1.9 |
| 0 | -0.2 | 74.82 | 2 | 3 | 1 | 1.38 |
| 0 | -3.31 | 139.84 | 7 | 2 | 0 | 1.91 |
| 0 | 3.05 | 73.45 | 3 | 0 | 3 | 1.47 |
| 0 | 2.47 | 180.55 | 4 | 0 | 2 | 2.41 |
| 0 | -0.86 | 241.33 | 14 | 1 | 2 | 1.76 |
| 0 | 0.62 | 219.9 | 9 | 3 | 1 | 1.45 |
| 0 | -1.53 | 154.27 | 6 | 1 | 2 | 1.6 |
| 0 | -1.63 | 177.02 | 7 | 0 | 0 | 5.42 |
| 0 | -1.04 | 279.43 | 15 | 2 | 2 | 1.44 |
| 0 | 1.34 | 117.89 | 5 | 1 | 2 | 1.62 |
| 0 | 0.56 | 74.96 | 3 | 2 | 0 | 1.47 |
| 0 | -9.92 | 425.21 | 16 | 5 | 0 | 1.26 |
| 0 | -3.74 | 164.75 | 7 | 1 | 0 | 2.87 |
| 0 | -1.21 | 54.37 | 5 | 0 | 0 | 4.03 |
| 0 | 4.64 | 17.07 | 1 | 1 | 1 | 1.93 |
| 0 | -0.31 | 165.95 | 6 | 3 | 1 | 1.78 |
| 0 | -2.57 | 96.92 | 5 | 2 | 0 | 1.98 |
| 0 | -1.32 | 238.17 | 15 | 0 | 2 | 2.26 |
| 0 | -0.84 | 238.34 | 10 | 2 | 2 | 1.34 |
| 0 | 4.93 | 163.72 | 8 | 2 | 1 | 1.74 |
| 0 | -1.65 | 297.92 | 15 | 5 | 1 | 1.06 |
| 0 | -1.53 | 154.27 | 6 | 1 | 2 | 1.6 |
| 0 | 2.63 | 110.31 | 2 | 1 | 2 | 1.47 |
| 0 | 2.76 | 163.11 | 8 | 0 | 0 | 5.54 |
| 0 | -0.94 | 223.32 | 12 | 5 | 1 | 1.05 |
| 0 | 0.61 | 132.76 | 3 | 2 | 1 | 1.54 |
| 0 | 2.33 | 173.25 | 8 | 2 | 1 | 1.76 |
| 0 | 3.22 | 80.67 | 5 | 0 | 1 | 2.8 |
| 0 | 1.43 | 163.11 | 8 | 0 | 0 | 5.47 |
| 0 | 0.2 | 152.99 | 4 | 2 | 1 | 1.5 |
| 0 | 5.34 | 25.78 | 1 | 1 | 3 | 1.39 |
| 0 | 2.76 | 163.11 | 8 | 0 | 0 | 5.54 |
| 0 | 1.74 | 137.01 | 4 | 0 | 2 | 1.72 |
| 0 | -4.17 | 169.86 | 5 | 3 | 0 | 1.71 |
| 0 | 0.17 | 199.84 | 9 | 1 | 1 | 2.22 |
| 0 | 2.44 | 110.31 | 2 | 1 | 2 | 1.41 |
| 0 | 1.28 | 137.01 | 4 | 0 | 2 | 1.7 |
| 0 | -1.46 | 214.32 | 12 | 0 | 0 | 6.36 |
| 0 | -4.1 | 186.37 | 8 | 2 | 0 | 2.15 |
| 0 | 6.5 | 37.3 | 1 | 0 | 0 | 2.92 |
| 0 | 1.88 | 114.81 | 6 | 0 | 1 | 2.66 |
| 0 | 2.54 | 114.81 | 6 | 0 | 1 | 2.77 |
| 0 | 3.54 | 140.26 | 5 | 0 | 1 | 2.88 |
| 0 | 2.23 | 131.88 | 7 | 0 | 1 | 2.87 |
| 0 | 2.89 | 131.88 | 7 | 0 | 1 | 2.96 |
| 0 | 5.11 | 140.11 | 6 | 0 | 1 | 2.84 |
| 0 | 3.89 | 157.33 | 6 | 0 | 1 | 2.96 |
| 0 | 5.12 | 123.04 | 5 | 0 | 1 | 2.75 |
| 0 | 4.25 | 157.33 | 6 | 0 | 1 | 3.04 |
| 0 | -0.7 | 197.25 | 10 | 0 | 1 | 2.93 |

logP: Octanol-water partition coefficient

PSA: Polar surface area

DC: Donor count

AlRC: Aliphatic ring count

ArRC: Aromatic ring count

BI: Balaban index
