# Supplementary material for: MLViS: A Web Tool for Machine Learning-Based Virtual Screening in Early-Phase of Drug Discovery and Development
Source: PLoS One. 2015 Apr 30;10(4):e0124600. doi: 10.1371/journal.pone.0124600 (PMC4415797; doi:10.1371/journal.pone.0124600)
Supplement: S2 Table — This data set contains 216 compounds and six molecular descriptors. (DOCX) [file pone.0124600.s002.docx]

**Table S2. Validation data set used in the study.** This data set contains 216 compounds and six molecular descriptors.

| group | logP | PSA | DC | AlRC | ArRC | BI |
| --- | --- | --- | --- | --- | --- | --- |
| 1 | -1,11 | 79,82 | 2 | 0 | 0 | 3,15 |
| 1 | 1,68 | 63,6 | 3 | 0 | 1 | 2,42 |
| 1 | 2,04 | 103,37 | 1 | 2 | 2 | 1,24 |
| 1 | 2,76 | 26,3 | 1 | 0 | 2 | 1,74 |
| 1 | 2,45 | 92,31 | 2 | 1 | 1 | 1,67 |
| 1 | 1,44 | 60,69 | 4 | 0 | 1 | 2,25 |
| 1 | 0,75 | 48,48 | 0 | 3 | 1 | 1,53 |
| 1 | 0,86 | 84,13 | 2 | 2 | 1 | 1,32 |
| 1 | 2,31 | 35,18 | 1 | 2 | 2 | 1,57 |
| 1 | 3,95 | 9,23 | 1 | 0 | 2 | 1,69 |
| 1 | -2,86 | 264,35 | 11 | 2 | 1 | 1,88 |
| 1 | 2,75 | 66,8 | 1 | 2 | 2 | 1,12 |
| 1 | 0,86 | 30,82 | 2 | 2 | 1 | 1,33 |
| 1 | 3,41 | 26,48 | 0 | 1 | 1 | 1,72 |
| 1 | 4,58 | 46,53 | 2 | 0 | 1 | 2,02 |
| 1 | 2,3 | 20,31 | 1 | 1 | 2 | 1,85 |
| 1 | 2,62 | 35,71 | 0 | 1 | 1 | 1,88 |
| 1 | -2,34 | 57,53 | 2 | 0 | 0 | 3,61 |
| 1 | 3,37 | 63,71 | 3 | 0 | 4 | 1,01 |
| 1 | 2,81 | 73,71 | 1 | 1 | 2 | 1,44 |
| 1 | 4,01 | 26,48 | 1 | 1 | 1 | 1,57 |
| 1 | 4,29 | 99,13 | 1 | 6 | 0 | 1,26 |
| 1 | 2,39 | 60,12 | 1 | 1 | 1 | 1,9 |
| 1 | 4,57 | 303,13 | 5 | 1 | 0 | 4,22 |
| 1 | 4,54 | 39,89 | 1 | 2 | 3 | 1,24 |
| 1 | 2,51 | 54,05 | 2 | 1 | 1 | 1,85 |
| 1 | 2,32 | 28,68 | 1 | 1 | 1 | 1,83 |
| 1 | 1,27 | 85,04 | 2 | 0 | 1 | 2,86 |
| 1 | 5,5 | 39,89 | 0 | 2 | 0 | 1,75 |
| 1 | 2,3 | 61,09 | 1 | 4 | 0 | 1,5 |
| 1 | 4,69 | 57,53 | 2 | 0 | 2 | 1,86 |
| 1 | 3,7 | 22,82 | 0 | 1 | 2 | 1,39 |
| 1 | 3,99 | 22,82 | 0 | 1 | 2 | 1,48 |
| 1 | 1,39 | 20,23 | 2 | 0 | 1 | 2,33 |
| 1 | 2,54 | 62,7 | 1 | 1 | 2 | 1,44 |
| 1 | 3,54 | 46,61 | 0 | 1 | 2 | 1,59 |
| 1 | 4,21 | 52,48 | 0 | 1 | 2 | 1,73 |
| 1 | 2,01 | 60,69 | 4 | 1 | 2 | 1,53 |
| 1 | 3,14 | 46,53 | 1 | 0 | 2 | 1,69 |
| 1 | 0,86 | 74,78 | 0 | 2 | 1 | 1,81 |
| 1 | 3,2 | 30,82 | 1 | 0 | 1 | 2,41 |
| 1 | 3,46 | 68,46 | 0 | 0 | 1 | 2,62 |
| 1 | 3,02 | 67,06 | 1 | 2 | 2 | 1,12 |
| 1 | 2,21 | 29,43 | 2 | 0 | 1 | 2,24 |
| 1 | 1,76 | 60,12 | 1 | 2 | 1 | 1,36 |
| 1 | 0,37 | 25,78 | 2 | 0 | 2 | 1,83 |
| 1 | 1,19 | 49,12 | 0 | 4 | 1 | 1,5 |
| 1 | 1,36 | 20,23 | 2 | 0 | 1 | 1,97 |
| 1 | 2,71 | 56,64 | 1 | 1 | 2 | 1,52 |
| 1 | 4,04 | 75,15 | 0 | 1 | 3 | 0,98 |
| 1 | 1,44 | 46,53 | 1 | 2 | 1 | 1,46 |
| 1 | 1,33 | 59,3 | 1 | 1 | 2 | 1,56 |
| 1 | 2,52 | 57,53 | 4 | 0 | 2 | 1,58 |
| 1 | 1,82 | 270,41 | 15 | 0 | 5 | 1,28 |
| 1 | 2,54 | 84,08 | 2 | 1 | 2 | 1,5 |
| 1 | 5,76 | 27,18 | 0 | 1 | 3 | 1,28 |
| 1 | 4,16 | 37,3 | 2 | 0 | 2 | 1,8 |
| 1 | 3,35 | 37,3 | 2 | 0 | 2 | 1,83 |
| 1 | 0,27 | 131,07 | 2 | 2 | 1 | 1,67 |
| 1 | 3,21 | 20,23 | 1 | 1 | 0 | 2,22 |
| 1 | 2,63 | 39,89 | 0 | 1 | 1 | 1,99 |
| 1 | -0,72 | 26,3 | 0 | 0 | 0 | 3,61 |
| 1 | 2,15 | 12 | 1 | 0 | 1 | 1,94 |
| 1 | 1,63 | 38,69 | 2 | 0 | 1 | 1,94 |
| 1 | 1,91 | 30,66 | 1 | 1 | 1 | 2 |
| 1 | 1,57 | 40,07 | 0 | 3 | 1 | 1,42 |
| 1 | 7,89 | 95,72 | 2 | 2 | 3 | 1,08 |
| 1 | 6,8 | 46,53 | 1 | 2 | 1 | 1,65 |
| 1 | 3,06 | 26,3 | 0 | 0 | 2 | 1,77 |
| 1 | 1,41 | 83,51 | 3 | 5 | 1 | 1,29 |
| 1 | 2,44 | 12,36 | 1 | 1 | 2 | 1,41 |
| 1 | 2,74 | 58,92 | 3 | 2 | 2 | 1,08 |
| 1 | 0,16 | 60,69 | 4 | 0 | 1 | 2,39 |
| 1 | 0,3 | 246,57 | 13 | 1 | 4 | 1,44 |
| 1 | 2,84 | 49,66 | 2 | 1 | 2 | 1,63 |
| 1 | 2,02 | 38,69 | 2 | 0 | 1 | 2,12 |
| 1 | 0,28 | 69,35 | 1 | 4 | 1 | 1,49 |
| 1 | -0,04 | 80,35 | 2 | 4 | 1 | 1,57 |
| 1 | -2,67 | 272,19 | 12 | 2 | 1 | 1,92 |
| 1 | 4,42 | 52,6 | 0 | 6 | 0 | 1,19 |
| 1 | 0,45 | 37,3 | 2 | 0 | 1 | 1,97 |
| 1 | 0,53 | 151,56 | 6 | 1 | 2 | 1,34 |
| 1 | 1,31 | 190,04 | 8 | 0 | 3 | 1,7 |
| 1 | 3,69 | 33,82 | 1 | 2 | 1 | 1,52 |
| 1 | -1,69 | 78,89 | 0 | 2 | 0 | 1,93 |
| 1 | 1,08 | 38 | 2 | 0 | 1 | 2,02 |
| 1 | 4,72 | 22,82 | 0 | 0 | 2 | 1,64 |
| 1 | 4,72 | 12 | 1 | 1 | 2 | 1,57 |
| 1 | 3,21 | 68,82 | 0 | 1 | 2 | 1,61 |
| 1 | -2,91 | 52,6 | 0 | 0 | 0 | 3,6 |
| 1 | 1,51 | 134,17 | 3 | 2 | 1 | 1,28 |
| 1 | 3,55 | 73,58 | 1 | 1 | 2 | 1,46 |
| 1 | 2,24 | 82,61 | 1 | 0 | 2 | 1,69 |
| 1 | 1,47 | 95,57 | 1 | 3 | 3 | 1,1 |
| 1 | 2,86 | 70,21 | 2 | 0 | 2 | 1,56 |
| 1 | 1,95 | 12,36 | 1 | 2 | 1 | 1,59 |
| 1 | 1,77 | 66,34 | 1 | 2 | 1 | 1,61 |
| 1 | 3,14 | 108,59 | 0 | 2 | 2 | 1,29 |
| 0 | 1,57 | 158,08 | 5 | 1 | 0 | 2,83 |
| 0 | -3,04 | 110,02 | 5 | 0 | 0 | 3,69 |
| 0 | -2,92 | 262,6 | 6 | 2 | 2 | 1,16 |
| 0 | -0,42 | 88,67 | 2 | 3 | 0 | 1,84 |
| 0 | -4,05 | 138,78 | 5 | 0 | 0 | 4,18 |
| 0 | -3,33 | 146,49 | 5 | 1 | 0 | 2,55 |
| 0 | 2,76 | 163,11 | 8 | 0 | 0 | 5,54 |
| 0 | -2,91 | 52,6 | 0 | 0 | 0 | 3,6 |
| 0 | -4,5 | 202,55 | 5 | 2 | 0 | 1,78 |
| 0 | 2,11 | 213,3 | 6 | 4 | 1 | 1,41 |
| 0 | -3,45 | 237,18 | 6 | 6 | 0 | 0,95 |
| 0 | -8,99 | 364,83 | 9 | 6 | 0 | 0,93 |
| 0 | 5,72 | 81,71 | 1 | 1 | 2 | 1,32 |
| 0 | 2,44 | 184,9 | 2 | 2 | 2 | 1,35 |
| 0 | 5,72 | 81,71 | 1 | 1 | 2 | 1,32 |
| 0 | -4,63 | 123,64 | 0 | 4 | 0 | 1,42 |
| 0 | -4,15 | 175,67 | 5 | 2 | 0 | 1,87 |
| 0 | -3,4 | 128,2 | 5 | 2 | 0 | 2,04 |
| 0 | -5,19 | 136,04 | 5 | 2 | 0 | 2,19 |
| 0 | -3,21 | 136,04 | 5 | 2 | 0 | 2 |
| 0 | -0,53 | 141,31 | 1 | 2 | 1 | 1,37 |
| 0 | -6 | 181,38 | 6 | 2 | 0 | 1,81 |
| 0 | -3,48 | 143,91 | 4 | 0 | 0 | 3,75 |
| 0 | 3,03 | 81,13 | 1 | 1 | 2 | 1,39 |
| 0 | 2,88 | 60,69 | 3 | 3 | 0 | 1,54 |
| 0 | 4,55 | 145,48 | 5 | 1 | 2 | 1,79 |
| 0 | 2,88 | 60,69 | 3 | 3 | 0 | 1,54 |
| 0 | -0,05 | 127,53 | 0 | 3 | 2 | 1,24 |
| 0 | 2,4 | 81,89 | 1 | 2 | 2 | 1,2 |
| 0 | -3,31 | 190,44 | 5 | 0 | 0 | 4,1 |
| 0 | -7,86 | 338,88 | 8 | 3 | 0 | 1,51 |
| 0 | 3,68 | 45,35 | 2 | 1 | 1 | 2,19 |
| 0 | 3,93 | 60,37 | 2 | 1 | 1 | 2,19 |
| 0 | 4,03 | 28,68 | 1 | 1 | 1 | 1,87 |
| 0 | -3,74 | 139,84 | 7 | 2 | 0 | 1,96 |
| 0 | -4,37 | 118,18 | 3 | 2 | 1 | 1,4 |
| 0 | 5,54 | 161,34 | 8 | 0 | 4 | 1,35 |
| 0 | 6,73 | 95,95 | 1 | 0 | 5 | 1,27 |
| 0 | -4,42 | 257,2 | 7 | 1 | 0 | 3,23 |
| 0 | -2,64 | 116,67 | 4 | 0 | 0 | 3,94 |
| 0 | -4,12 | 199,52 | 6 | 2 | 0 | 1,85 |
| 0 | 5,97 | 112,07 | 3 | 1 | 2 | 1,57 |
| 0 | 3 | 146,36 | 3 | 1 | 4 | 1,36 |
| 0 | 5,52 | 112,22 | 1 | 1 | 4 | 1,34 |
| 0 | -4,09 | 209,76 | 10 | 3 | 0 | 1,59 |
| 0 | -8,78 | 388,29 | 17 | 5 | 0 | 1,12 |
| 0 | -2,39 | 127,45 | 5 | 1 | 0 | 2,5 |
| 0 | -6,59 | 369,58 | 13 | 3 | 0 | 1,97 |
| 0 | 5,7 | 108,59 | 2 | 1 | 4 | 1,04 |
| 0 | -1,79 | 100,05 | 2 | 0 | 0 | 3,39 |
| 0 | 0,35 | 87,74 | 3 | 2 | 1 | 1,56 |
| 0 | 2,47 | 72,83 | 3 | 2 | 1 | 1,71 |
| 0 | 5,17 | 126,52 | 5 | 1 | 3 | 1,45 |
| 0 | 4,63 | 133,24 | 5 | 1 | 3 | 1,46 |
| 0 | 1,64 | 44,1 | 2 | 1 | 1 | 1,48 |
| 0 | -5,54 | 290,8 | 8 | 3 | 0 | 1,48 |
| 0 | -0,75 | 49,66 | 2 | 1 | 0 | 2,32 |
| 0 | 6,58 | 79,84 | 2 | 0 | 4 | 1,13 |
| 0 | 1,3 | 139,32 | 3 | 0 | 2 | 1,61 |
| 0 | -4,41 | 205,97 | 5 | 3 | 0 | 1,54 |
| 0 | -5,05 | 318,3 | 4 | 5 | 0 | 1,33 |
| 0 | 2,25 | 109,42 | 3 | 0 | 2 | 1,7 |
| 0 | -8,45 | 388,29 | 17 | 5 | 0 | 1,1 |
| 0 | 6,71 | 126,17 | 7 | 1 | 4 | 1,14 |
| 0 | -3,84 | 82,83 | 5 | 1 | 0 | 2,3 |
| 0 | -3,84 | 82,83 | 5 | 1 | 0 | 2,3 |
| 0 | 5,37 | 53,09 | 2 | 0 | 4 | 1,46 |
| 0 | 3,12 | 111,9 | 5 | 0 | 3 | 1,47 |
| 0 | 2,89 | 127,69 | 6 | 0 | 4 | 1,25 |
| 0 | -0,36 | 152,08 | 11 | 0 | 0 | 5,98 |
| 0 | -0,25 | 127,15 | 2 | 2 | 1 | 1,49 |
| 0 | -4,68 | 193,68 | 5 | 0 | 0 | 3,72 |
| 0 | 5,13 | 119,99 | 5 | 0 | 4 | 1,63 |
| 0 | 2,36 | 112,65 | 4 | 1 | 2 | 1,58 |
| 0 | 1,76 | 132,88 | 5 | 1 | 2 | 1,72 |
| 0 | 2,76 | 112,65 | 4 | 1 | 2 | 1,53 |
| 0 | 3,79 | 99,76 | 4 | 1 | 3 | 1,31 |
| 0 | 5,5 | 99,76 | 4 | 0 | 4 | 1,63 |
| 0 | 4,34 | 142,28 | 5 | 0 | 4 | 1,5 |
| 0 | 3,72 | 142,28 | 5 | 0 | 4 | 1,52 |
| 0 | 2,41 | 87,62 | 1 | 2 | 1 | 1,51 |
| 0 | 1,24 | 132,91 | 3 | 0 | 0 | 3,77 |
| 0 | 2,4 | 64,8 | 4 | 2 | 2 | 1,34 |
| 0 | -3,08 | 182,34 | 5 | 3 | 0 | 1,51 |
| 0 | -3,15 | 162,11 | 5 | 3 | 0 | 1,51 |
| 0 | -3,54 | 162,11 | 5 | 3 | 0 | 1,55 |
| 0 | -5,95 | 271,27 | 8 | 3 | 0 | 1,5 |
| 0 | 3,68 | 59,9 | 0 | 4 | 2 | 1,11 |
| 0 | 1,53 | 99,09 | 3 | 3 | 1 | 1,31 |
| 0 | 3,83 | 114,99 | 5 | 3 | 2 | 1,16 |
| 0 | -2,73 | 98,74 | 4 | 2 | 0 | 2,19 |
| 0 | 1,88 | 80,92 | 5 | 1 | 0 | 2,21 |
| 0 | -0,41 | 131,61 | 6 | 1 | 1 | 1,75 |
| 0 | -3,86 | 274,15 | 6 | 2 | 1 | 1,82 |
| 0 | 3,71 | 69,85 | 2 | 3 | 2 | 0,99 |
| 0 | 3,79 | 70,26 | 1 | 5 | 2 | 0,93 |
| 0 | 3,68 | 94,27 | 1 | 2 | 1 | 1,49 |
| 0 | -0,71 | 77,76 | 5 | 1 | 1 | 1,61 |
| 0 | -0,57 | 91,35 | 4 | 1 | 1 | 1,86 |
| 0 | 2,46 | 130,33 | 2 | 1 | 2 | 1,68 |
| 0 | 1,37 | 124,01 | 3 | 4 | 1 | 1,1 |
| 0 | 1,67 | 57,53 | 2 | 0 | 1 | 2,3 |
| 0 | 3,01 | 71,2 | 3 | 1 | 2 | 1,4 |
| 0 | 3,21 | 88,52 | 3 | 2 | 3 | 1,02 |
| 0 | 4,13 | 65,92 | 2 | 2 | 4 | 1 |
| 0 | 1,91 | 83,83 | 2 | 5 | 0 | 1,47 |
| 0 | -11,41 | 459,42 | 12 | 1 | 0 | 3,94 |
| 0 | 1,41 | 82,69 | 3 | 0 | 2 | 1,77 |
| 0 | 2,26 | 66,42 | 3 | 3 | 2 | 1,17 |
| 0 | -2,45 | 155,13 | 4 | 0 | 1 | 2,76 |
| 0 | -1,37 | 121,31 | 4 | 0 | 1 | 2,41 |
| 0 | 1,15 | 91,85 | 3 | 0 | 2 | 1,68 |
| 0 | -1,53 | 125,67 | 3 | 0 | 1 | 2,54 |
| 0 | -0,63 | 91,85 | 3 | 0 | 1 | 2,37 |
| 0 | -0,79 | 91,85 | 3 | 0 | 1 | 2,19 |
| 0 | -2,2 | 83,12 | 2 | 0 | 0 | 3,2 |
| 0 | 2,81 | 93,77 | 2 | 3 | 3 | 0,88 |
| 0 | 0,14 | 159,82 | 5 | 3 | 2 | 1,48 |

logP: Octanol-water partition coefficient

PSA: Polar surface area

DC: Donor count

AlRC: Aliphatic ring count

ArRC: Aromatic ring count

BI: Balaban index
